# StreetArt4Sustainability dataset: Mapping aesthetic emotions to street art

**DOI:** 10.3758/s13428-026-03119-5

**Published:** 2026-07-24

**Authors:** Patrícia Arriaga, Erin M. Buchanan

**Affiliations:** 1 Iscte-University Institute of Lisbon, CIS-ISCTE, Lisbon, Portugal; 2 Harrisburg University of Science and Technology, Harrisburg, PA USA

**Keywords:** Street art, Dataset, Affective responses, Self-transcendent emotions, Epistemic emotions, Negative emotions, Interest in street art, Sustainability consciousness

## Abstract

A novel dataset of 556 street art images is presented, accompanied by affective evaluations from 1,239 Portuguese and Brazilian participants. Artworks were selected to reflect themes associated with the United Nations Sustainable Development Goals. Using a stimulus-sampling design, each participant completed an online survey in which 10 randomly selected artworks were presented and reported their responses in terms of valence, arousal, and specific emotion labels (being moved, awe, inspiration, hope, sadness, fear, anger, emotional connection, reflection, awareness, and interest), as well as their interest in street art and sustainability consciousness. Multilevel analyses showed that higher interest in street art and greater sustainability consciousness were consistent predictors of more positive emotional responses to the artworks. In contrast, the effects of gender and age were negligible, and national differences emerged only for feeling moved and awe. Network analyses revealed a highly interconnected emotional structure, with three clusters: self-transcendent, epistemic, and negative emotions. Feeling moved and emotional connection occupied central bridging positions, showing both direct and indirect links across positive and negative emotion clusters. Overall, these findings indicate that street art evokes a broad range of interconnected self-transcendent, cognitive-epistemic, and negative emotions, highlighting the complexity of viewers’ responses to the artworks. The dataset provides a valuable resource for research on emotional responses to street art and can support broader investigations into visual perception, aesthetic processing, and the communication of sustainability-related themes.

## StreetArt4Sustainability dataset: Mapping aesthetic emotions to street art

Street art has emerged over the past 50 years as a distinctive artistic form that combines aesthetic expression with social and political commentary (Gonçalves & Milani, 2022). Once considered an illicit practice, its acceptance and popularity have grown, establishing it as a relevant cultural expression since the 2000s (Bengtsen, 2016). Nowadays, many public and private spaces are commissioned for street art creation, with many artworks making their way to auctions (Gonçalves & Milani, 2022). Several initiatives (e.g., festivals, competitions) also promote street art, with community-driven street art projects often engaging residents and attracting tourists and online audiences, thereby enhancing communities’ cultural diversity (MacDowall, 2019). In this study, we use the umbrella term “street art” because it encompasses diverse styles displayed across a broad range of locations, rather than using other restricted terms such as urban art or graffiti (Gartus & Leder, 2014).

Street art has been defined as “self-authorized images, characters, and forms created in or applied to surfaces in the urban space that intentionally seek communication with a larger circle of people” (Blanché, 2015, p. 33). This definition highlights its variety, from simple graffiti letters to elaborate murals, typically found in public places, including outdoor and indoor surfaces and abandoned factories (Blanché, 2018). It also underscores street artists’ communicative purpose to engage a broad audience and amplify their message and impact (Curtis, 2009). Street artists often depict local community values (Bacharach, 2015) and use their art to beautify spaces (Visconti et al., 2010), but most frequently, they use it to express social and political messages (Bacharach, 2015; Glăveanu, 2017; Ross et al., 2020), including many of the social, environmental, and economic challenges that are considered essential for several global organizations. Recently, the potential of street art to support the United Nations (UN) 2030 Agenda for Sustainable Development has gained recognition (Baily, 2019; Bengtsen, 2018; Hansen, 2022). Despite widespread commitment to using street art as a tool for social change, research into its impact on citizens is scarce. This gap highlights the need to examine how street art elicits emotions and reflective thinking about its message (Sommer & Klöckner, 2021). Existing research on the influence of various visual stimuli, including visual art, on human attention, emotions, cognitions, and behaviors underscores the importance of this area of study.

### The need for a dedicated dataset about street art

The development of databases of standardized emotion-eliciting images has advanced research, particularly in understanding their impact on capturing viewers’ attention and triggering emotions. A recent searchable database of emotional stimuli datasets, designated as KAPODI, was created to compile research stimuli published since 1963 (Diconne et al., 2022). The database categorizes stimuli by type, including images, faces, videos, audio, words, and combined modalities. Among the image-based datasets listed, the stimuli span a wide range of content, including objects, food, natural scenes, threat scenarios, and social contexts, and the repository remains open, allowing the inclusion of newly developed datasets. Emotional ratings of valence (i.e., the degree of displeasure–pleasure or negativity–positivity experienced) and arousal (i.e., the degree of calm–excitement experienced) are systematically reported in the majority of datasets, grounded in the dimensional approach to affective experience (Posner et al., 2005; Russell, 1980). Only a few follow the categorical approach to evaluate specific affective states. However, among the 38 image datasets currently available, none specifically pertains to artworks, underscoring the need for dedicated resources such as the present dataset.

Research involving artwork images is indeed less common, and such studies often rely on custom-prepared images tailored for specific experimental goals, requiring pretesting of the material (Gartus & Leder, 2014; Vessel et al., 2012, 2013, 2014). This approach presents several challenges, including a lack of diversity and reduced study replicability, underscoring the benefits of developing and analyzing ratings of a large set of stimuli. Nevertheless, some artwork datasets have already been created. For example, the ArtEmis dataset (Achlioptas et al., 2021) includes a large collection of paintings and artistic photographs selected from WikiArt, which were evaluated in terms of the dominant emotion experienced. The JenAesthetics dataset (Amirshahi et al., 2015) includes Western paintings from well-known artists spanning the fifteenth to twentieth centuries, with subjective ratings of aesthetic value, beauty, liking, and familiarity with the artist and artwork. The Vienna Art Picture System (VAPS; Fekete et al., 2023) offers normed fine-art paintings across different historical periods, styles, and genres, with ratings of emotional valence and arousal, liking, visual complexity, and familiarity. The Art500k dataset (Mao et al., 2017) was developed for large-scale visual art analysis in computer vision and contains artworks annotated with metadata, such as artist, genre, art movement, and other labels to support artwork classification. Finally, the art.pics dataset (Thieleking et al., 2020) presents art images generated by a deep learning algorithm, intended to be less complex than traditional paintings and to integrate styles of various artists with common images of animals, plants, and objects.

The public, contextual, and culturally situated nature of street art underscores the need for a specialized dataset, which, to our knowledge, has not yet been created. This dataset would support the development of theoretical frameworks tailored to understanding the aesthetic responses elicited by this unique art form. Like other forms of visual stimuli, street art images are expected to elicit a range of emotional responses in viewers (Branco et al., 2023; Huang et al., 2015), depending on the artwork’s content and context. By visually representing relevant issues, street art may raise awareness of the topics it depicts, with potential cultural, educational, and social impacts. Deconstructing the artwork’s meaning and the artist’s intent can be relevant for stimulating critical thinking and action (Curtis, 2009; Opermanis et al., 2015). This emotional–cognitive engagement may also make street art memorable and impactful for viewers (Arce-Nazario, 2016; Baldwin & Chandler, 2010). Thus, our work involved creating a dataset of street art images to enhance scientific knowledge of its effects on viewers and to present data on its emotional impact to support future research.

Among the several motivations for creating this dataset, we highlight the following: First, street art, unlike art forms traditionally confined to galleries or museums, is an artistic expression born in the streets, making it accessible to a broad community, although artworks are often restricted to specific community spaces, vulnerable to change or disappearance over time due to factors such as weather, the creation of new art over them, or destruction/vandalism. Therefore, creating a photographic dataset of street artworks also preserves artists’ work against potential loss (Blanché, 2018). Secondly, research has extensively documented the picture-superiority effect compared to textual communication across various age groups, in both immediate and delayed recall (Ally et al., 2008; Whitehouse et al., 2006). Visual images tend to be processed more efficiently and remembered better than words, indicating their high attention-capturing potential (McBride & Dosher, 2002). Furthermore, when they carry emotional content, they often elicit earlier and stronger emotional responses than words (Houwer & Hermans, 1994; Margalhos et al., 2019), indicating that the use of visual stimuli is very effective in producing an impact on humans, with several datasets being developed to study its effects on cognition, emotions, and behavior systematically. Research also suggests that combining textual and visual elements may enhance message comprehension and retention, particularly when they are congruent (Powell et al., 2015). Our dataset also includes artworks featuring verbal elements, which may provide greater contextual understanding and reduce subjectivity in interpreting the artist’s narrative intent. Thirdly, our dataset comprises street art images from various countries, created by artists with diverse styles, capturing a wide range of issues and cultural perspectives.

### Affective responses to street art

In examining affective responses to visual artworks, research has typically focused on valence and arousal dimensions (Russell, 1980; Russell & Carroll, 1999) and, occasionally, on some discrete emotions, most often using a categorical approach based on Ekman’s theory of basic emotions (Ekman, 1994; Ekman & Friesen, 1971). Because Ekman’s theory was initially developed to investigate the universality of facial expressions, it relies on a limited set of emotions, focusing more on negative than positive emotions (Branco et al., 2023), and may not adequately capture the complexity of emotions in relation to art. While other studies have explored general aesthetic evaluations, such as liking (Gerger et al., 2014), only a few studies have extended to aesthetic emotions such as *awe*, *being moved*, *inspiration*, and *interest* (e.g., Hager et al., 2012; Hagtvedt et al., 2008; Konečni, 2015). Furthermore, street art conveys messages intended to provoke purpose-oriented action. Thus, in this work, we also incorporated epistemic, reflective, and theme-directed feelings, as well as negative emotions such as fear, sadness, and anger (Schindler et al., 2017).

Drawing on theoretical frameworks that posit that emotions energize and mobilize action, we adopt a functionalist perspective that holds that emotions serve specific, often adaptive purposes. In this view, an emotion is considered adaptive when it produces changes that lead to better outcomes for the individual (Karnaze & Levine, 2018; Lench et al., 2016) and for interpersonal interactions (Keltner & Haidt, 1999), including the shaping of collective identities, intergroup behaviors, and cultural values and norms (de Rivera, 2014; Fischer & Manstead, 2008). The affective responses assessed in this study are directly related to the selection of street artworks, which depict a diverse range of themes but mainly address environmental, economic, and social challenges.

First, by emphasizing the urgency of social and environmental issues or depicting the consequences of inaction, the artworks may trigger negative emotions, such as *sadness*, *fear*, or *anger*. *Sadness* is often triggered by perceived losses, powerlessness, or unmet goals (Huron, 2018; Karnaze & Levine, 2018; Oatley & Duncan, 1994; Zickfeld et al., 2020), which may lead to rumination or withdrawal, but may also motivate actions like politeness and generosity (Frijda, 1986; Small & Lerner, 2008), and encourage individuals to ponder on the causes and consequences, contributing to more systematic and detailed problem-solving about the stimuli depicted (Andrews & Thomson, 2009; Lyubomirsky et al., 1998). *Fear* is commonly linked to identifiable threats, triggering a fight-or-flight response to protect individuals from immediate harm (Nesse & Ellsworth, 2009). Still, it can motivate precautionary actions (Parsafar & Davis, 2018) and promote the adoption of recommended behaviors, thereby reducing risk-taking and risk-related behaviors (Tannenbaum et al., 2015). *Anger* typically arises from perceived injustice, directing attention toward ethical and moral concerns such as human rights and justice, which may drive individuals to overcome these challenges. It may also occur when an artwork is appraised as incongruent with personal values (Silvia & Brown, 2007). Thus, as an approach-oriented emotion, anger can motivate viewers to engage with the issues depicted and has been identified as a driving force in activism (Iyer et al., 2007; Yang, 2000), political actions (van Troost et al., 2013), and other forms of proactive actions, such as aiding the disadvantaged, supporting wealth redistribution policies, engagement in anti-poverty initiatives, and petition signing against abuse and mistreatment (Batson et al., 2007; Montada & Schneider, 1989; Thomas et al., 2009; Wakslak et al., 2007). Therefore, we included these negative emotions because they can also be relevant to understanding how art can motivate action on the issues portrayed.

Second, the artworks may evoke positive emotions typically linked to aesthetic experience. As posited by the broaden-and-build theory of positive emotions (Fredrickson, 2001, 2013), experiencing positive emotions can have more than an immediate “feel-good” function by “broadening” or widening the scope of our attention, cognition, and action, which can “build” long-term personal and social resources. For this study, we focused on prototypical aesthetic emotions also categorized as “self-transcendent,” such as *awe*, *inspiration*, and *moved*, because they are often elicited by situations or stimuli that broaden individuals’ perspectives by shifting their focus outward, giving rise to “other-focused appraisals” (Stellar et al., 2017). *Awe* is commonly triggered by perceptions of vastness and a need for cognitive accommodation (Keltner & Haidt, 2003), which tend to motivate contemplation and submission. *Inspiration* is typically elicited when witnessing portrayals of moral beauty or excellence, including unexpected gestures of kindness and compassion (Sparks et al., 2019), whereas being *moved* typically arises from sudden communal sharing, often motivating social connection (Cullhed, 2020; Zickfeld et al., 2019). These other-oriented appraisals enhance an individual’s awareness, sensitivity, and responsiveness to others (Pizarro et al., 2021). Therefore, such emotions tend to impact personal experiences, but also foster social connections, reduce stereotypes, and increase prosocial collective action (Krämer et al., 2017; Pizarro et al., 2021; Stellar et al., 2017). Relatedly, our study also included the emotion of *hope*, which is related to moral elevation (Keltner & Haidt, 2003; Pohling & Diessner, 2016) and is often triggered by stimuli that portray a positive outlook for the future, demonstrate perseverance in overcoming obstacles, or depict encouragement (Dale et al., 2023). This emotion may also shape resilience, interconnectedness, proactive economic behavior, and environmentally sustainable practices, motivating individuals to strive for a more optimistic future (Fredrickson, 2013; Reichard et al., 2013).

Finally, we assessed epistemic and theme-directed emotional responses. Artworks may prompt viewers to reflect on their content and its broader implications, enhancing awareness and cognitive engagement with the depicted themes. Epistemic emotions include feelings of *interest* arising from appraisals of novelty, complexity, uncertainty, or ambiguity, which can foster exploration, learning, and both individual and collective well-being (Kashdan & Silvia, 2009; Morton, 2010; Phillips et al., 2015; Schindler et al., 2017; Vogl et al., 2021; von Stumm et al., 2011). Metacognitive feelings, in turn, refer to affective experiences that provide insight into cognitive processes, such as feelings of comprehension, awareness, or difficulty (Nerantzaki et al., 2021; Vogl et al., 2021). In this study, we examined how the artwork evokes *reflection, awareness,* and *emotional connectedness* with the depicted themes, considering that these responses may involve both epistemic and metacognitive components: epistemic, through the need for further understanding, and metacognitive, through *awareness* and one’s *connection* with the theme (Sommer & Klöckner, 2021). These labels were treated as subjective emotional responses with epistemic and cognitive theme-directed components.

### Predictors of emotional response to artworks

Emotions elicited by the artworks may also vary according to cultural and individual factors, reflecting the diversity of viewers’ reactions and interpretations.

*Culture* may affect how visual stimuli are perceived, potentially contributing to differences in emotional responses (Barke et al., 2012; Soares et al., 2015). Although nationality is a proxy for broader cultural influences, prior work has shown that people from different countries can respond differently to affective images (Huang et al., 2015; Soares et al., 2015). For example, in a Portuguese validation study of the International Affective Picture System (IAPS, Lang et al., 2005), Soares et al. (2015) found that Portuguese participants reported lower levels of valence and arousal than those described in the Brazilian norms for these images (Lasaitis et al., 2008; Ribeiro et al., 2005).

*Gender* comparisons are also frequently considered in many dataset validations (Diconne et al., 2022), with studies suggesting that men tend to react more positively to certain pleasant stimuli (e.g., erotic), and women often exhibit a negative bias, responding with lower valence and higher arousal to negative stimuli. This pattern is evidenced in various cultural contexts (Barke et al., 2012; Biele & Grabowska, 2006; Bradley et al., 2001a, 2001b), including Portuguese and Brazilian samples (Lasaitis et al., 2008; Soares et al., 2015). In relation to aesthetic beauty appreciation of art through subjective and neurological responses, Cela-Conde et al. (2009) found that men and women showed no differences in frequency or subjective ratings. However, men showed stronger neural activity in certain brain areas after 300 ms of viewing images they found more beautiful, suggesting a focus on global features, whereas women may integrate global and local features in their beauty assessments, but the precise cognitive mechanisms underlying gender differences in beauty perception remain unclear.

*Age* can be another relevant predictor. A systematic review using the IAPS database has shown that older adults typically find images more arousing than younger adults (Branco et al., 2023), suggesting a “positivity bias” as people age by prioritizing positive content and perceiving negative images in a more positive light (Branco et al., 2023). Therefore, if street art conveys a positive message, older adults might respond more favorably and intensely than younger individuals. Variations in responses may also arise from life experiences and personal beliefs about street art. Older generations, for whom street art was less mainstream and often linked to youthful rebellion, might have stronger negative reactions. In contrast, younger generations, having grown up in an era where street art is more prevalent and culturally accepted, might view it as a legitimate form of artistic expression, making them more receptive and likely to respond with positive emotions.

Past research has also shown that an individual’s *general interest in art* can be a significant predictor of emotions toward artistic expressions (e.g., Gartus & Leder, 2014), which relate to engagement and appreciation for various art forms. Those with greater interest may also be more familiar with the principles of art, enabling them to connect with other works and be more attentive to the visual properties, intended message, and emotional tones in an artwork. Consequently, their emotions may be more intense and varied based on their prior exposure, knowledge, and motivation, as well as the context in which the artwork was created and the sociopolitical message. While some individuals view street art as a transformative artistic expression in public spaces, others might perceive it as vandalism (Gartus & Leder, 2014), suggesting that a general interest in art might be distinct from the specific interest in street art. Gartus and Leder (2014) found a positive relationship between appreciation of general art and street art, suggesting related yet distinct forms of appreciation, and noted that greater interest in street art was associated with higher emotional and cognitive evaluations of the artworks. Thus, in this study, we will explore whether interest in street art predicts the emotions elicited by street art images.

Finally, given that many artworks focus on themes related to the three pillars of sustainable development, we will examine whether an individual’s *sustainability consciousness* is associated with their emotional responses. While no studies directly address this relationship, it is plausible that greater awareness of sustainability concerns could lead to stronger, more intense emotional reactions to artworks that either resonate with or challenge their values.

### Research aims

#### Dataset creation

Our primary goal was to develop a StreetArt4Sustainability dataset for research purposes by examining subjective emotional experiences toward street artworks.

#### Dataset validation

To validate the dataset, we examined whether the affective responses elicited by the artwork images reproduced patterns reported in prior research on emotion. Drawing on complementary theories and conceptualizations of emotion, we considered both dimensional and categorical approaches, analyzing valence and arousal as well as specific emotion labels. Additionally, we examined whether interest in street art (ISA), sustainability consciousness (SC), and sociodemographic factors contributed to explaining affective responses and the relationships among these feelings. Regarding the dimensional model, the relationship between valence and arousal is a matter of debate, with various hypotheses proposed (e.g., independence; positive or negative linear relation; V-shaped relationships), also suggesting that their relation depends on context (e.g., Kuppens et al., 2017; Nandy et al., 2023; Yik et al., 2023). When using images as stimuli, some studies show a curvilinear relationship where both high positive and negative valences correlate with higher arousal, particularly when using image sets containing biologically salient content related to survival or reproduction, which have been found to be less context-dependent (Bradley et al., 2001a, 2001b; Branco et al., 2023; Lasaitis et al., 2008; Paes et al., 2016; Soares et al., 2015). However, this pattern may not generalize uniformly across image types. Socially meaningful and symbolically complex stimuli may require more elaborative interpretation, making the relation between valence and arousal less direct (Sakaki et al., 2012). In line with this view, Kuppens et al. (2013) found that, in responses to nonfigurative modern art paintings, arousal was best modeled as an asymmetric V-shaped function of valence, with stronger increases in arousal for positive than for negative valence, although this relation was weak. Similar reasoning may apply to specific emotional responses, such as being moved and awe, which may be experienced in both positively and negatively valenced forms (e.g., being joyfully or sadly moved; experiencing awe in response to beauty or threat) (Keltner & Haidt, 2003; Menninghaus et al., 2015; Schindler et al., 2022). Likewise, responses such as interest, reflection, awareness, and emotional connection to the themes may also vary as a curvilinear function of experienced valence, because artworks felt as strongly positive or strongly negative may be more likely to invite perceived relevance and interpretative engagement (Leder et al., 2004). Accordingly, we tested both linear and quadratic models predicting arousal and each affective response from valence. Additionally, we modeled each affective response as a linear function of felt arousal to explore whether higher subjective activation in response to the images would be associated with stronger affective responses.

Finally, we considered that the specific emotion labels would systematically co-occur, an assumption supported by cross-cultural studies that have favored network theory (e.g., Lange & Zickfeld, 2023). Based on this approach, we explored how specific emotions were interrelated, examining whether they formed distinct clusters (Schindler et al., 2017) and how they were connected across clusters.

The project, including the anticipated studies, was preregistered on AsPredicted, and the general aims presented in this study are available at https://aspredicted.org/hhzc-7h8v.pdf.

## Method

### Participants

A sample of 1,610 participants was initially recruited through an online survey, using a non-probabilistic, convenience sampling strategy via (i) social media (*n* = 723; 44.9%), (ii) the Clickworker crowdsourcing platform in exchange for a small payment of €3 to each participant (*n* = 705; 43.8%), and (iii) a university pool of students in exchange for course credit (*n* = 182; 11.3%). A total of 371 participants were removed because they either did not respond or failed the attention check (*n* = 346) or did not have either Portuguese or Brazilian nationality (*n* = 25). A completion rate of 76.96% was achieved, yielding a final sample of 1,239 participants. As shown in Table 1, the sample comprised 56% women (*n* = 694) and 43.7% men (*n* = 542), aged between 18 and 76 years (*M* = 34.64, *SD* = 12.05), 51.8% Brazilian, 47.1% Portuguese, and only 1% (*n* = 13) with dual nationality. Most resided in Portugal (50.4%) or in Brazil (49.2%). The highest educational attainment was high school education (10th–12th grade; 38.7%) or a bachelor’s degree (33.9%). Concerning marital status, the majority were single (53.4%). The majority indicated that they were “coping” or “living comfortably” with their current income (76.6%) and reported a medium social status. Finally, most participants were never involved in any artistic profession or activity (62.6%).

**Table 1:** Descriptive statistics for sociodemographic variables

Variables	*n* (%)	Variables	*n* (%)
Gender		Marital Status	
Women	694 (56.0)	Single	662 (53.4)
Men	542 (43.7)	Married/Cohabiting	502 (40.5)
Other	3 (0.2)	Divorced/Widowed	76 (5.9)
Nationality		Residency	
Portuguese (PT)	584 (44.1)	Portugal	625 (50.4)
Brazilian (BR)	642 (51.8)	Brazil	610 (49.2)
Both PT & BR	13 (1.0)	Other	5 (0.4)
Education (concluded)		Art-related profession/activities	
Elementary (≤ 9th grade)	22 (1.8)	Never	775 (62.6)
High (10th – 12th grade)	480 (38.7)	Past	263 (21.2)
Bachelor/Graduation	420 (33.9)	Currently (part-time)	163 (13.2)
Pos-graduation/Master	278 (22.4)	Currently (full-time)	30 (2.4)
Doctorate	23 (1.9)		
Socio-economic Status (subjective)			*M* (*SD*)
Living comfortably	354 (28.6)	Age (18–16 years)	34.64 (12.05)
Coping	597 (48.2)	Subjective social status (0–10)	5.73 (1.67)
Difficult	204 (16.5)		
Very Difficult	72 (5.8)		

### Street art stimuli

We selected the street artworks using the following criteria: (i) encompassing a diverse range of styles and techniques (e.g., 2D or 3D paintings, stencils, graffiti, reverse graffiti, wheat pasting, stickers, posters) photographed in different visual art perspectives and angles; (ii) depicted on walls (e.g., buildings, skyscrapers, houses or facilities; garage doors, rolling gates; sides of passageways such as alleys, lanes, tunnels, underground or elevated train stations; fences; utility boxes, public installations; agricultural structures, such as grain silos or water towers) or on horizontal surfaces (e.g., sidewalks, public squares/plazas, rooftops); and (iii) placed in diverse locations, such as urban, suburban, rural, natural environments (e.g., mountain, desert, coastal areas), transit points (e.g., train stations, roadside houses). Exclusion criteria included nonpictorial artworks, such as street acts or performances (e.g., street theater, clown acts, human statues), or digital works. Most images were selected from the Internet by searching for street art across different sources (e.g., Google), social media platforms (e.g., Instagram, Facebook, Pinterest, Twitter), Flickr, street art blogs, and dedicated street art websites and apps. We asked artists and/or photographers for authorization to use their copyrighted material. Once we received permission, we incorporated additional artworks from these artists. During this collection process, we also selected images of street art from websites that stated they were available for reuse and modification.

A total of 556 street art images were selected. All images were edited to maintain uniform dimensions (1,024 pixels wide by 768 pixels high) and to adhere to a 4:3 aspect ratio, as in prior datasets (e.g., IAPS; Lang et al., 2005). To ensure all images had exact dimensions, each was centered against a black background. The dataset includes images from seven world regions and 48 countries or locations of creation. Europe is the most represented region (302 images from 23 countries/locations), with Portugal (*n* = 101), Spain (*n* = 45), and France (*n* = 40) being the most represented. North America follows with 117 artworks from three countries: the United States (*n* = 82), Mexico (*n* = 29), and Canada (*n* = 6). In South America, 80 images come from six countries, mainly from Brazil (*n* = 42) and Colombia (*n* = 25). Asia is represented by 26 images from six countries/locations, and Africa by 11 images from three countries. Oceania contributes 11 images from two countries/locations, mostly Australia (*n* = 10). Finally, Central America and the Caribbean are represented by nine images from five countries, mainly El Salvador (*n* = 5).

### Measures

Measures were translated into Portuguese using forward and backward translation procedures. Then, Portuguese and Brazilian students screened the final translated versions to ensure comprehension among their respective nationalities.

*Sociodemographic variables* included age, gender, nationality, country of residence, educational level, marital status, professional artistic/cultural activities, subjective social status (SSS), and perceived socioeconomic status (SES). For SSS, the MacArthur Scale of Subjective Social Status (Adler et al., 2000; Giatti et al., 2012) was used with a 10-step ladder where participants reported the number (between 0 and 10) that best characterized their current situation within their country (0 = “people who are in the worst condition or the lowest standing in terms of income, education level, job/occupation prestige or unemployment”; 10 = “people in the best situation or highest standing in society”). For SES, we assessed household income using the Portuguese version of the ESS Round 10 (European Social Survey European Research Infrastructure, 2020), with four response options ranging from 1 (“living comfortably on present income”) to 4 (“very difficult on present income”).

*Emotional responses* were assessed using a list of labels informed by prior studies, with the number of items kept to a minimum to minimize participant fatigue, while acknowledging that artworks can elicit a wide range of affective reactions. Two core dimensions of emotional experience were assessed according to the circumplex model of emotions (Russell, 1980): *arousal* (1 = “very calm” to 9 = “very aroused”) and hedonic *valence* (1 = “high displeasure/negative” to 9 = “high pleasure/positive”). Participants also rated 11 specific affective responses (1 = “none” to 9 = “very much”) introduced with the common stem “To what extent does the image make you feel…” Eight emotions, derived from Fredrickson’s modified Differential Emotions Scale (mDES; Fredrickson, 2001, 2013), were assessed using two labels for each emotion: “*moved/touched*,” *“hopeful/optimistic*,” “*awe/amazed*,” “*inspired/elevated*,” “*interested/curious*,” *“scared/afraid*,” “*sad/unhappy*,” and “*angry/outraged*.” Three ad hoc items were added to assess responses directed specifically toward the artwork’s content: “*emotionally connected to the theme*,” “*reflect on the theme*,” and “*become aware and alert to the theme*.” This list included self-transcendent aesthetic emotions, namely *awe, moved,* and *inspiration* (Pizarro et al., 2021; Schindler et al., 2017), *hope,* given its link with transcendent emotions and moral elevation (Keltner & Haidt, 2003; Pohling & Diessner, 2016), negative emotions often elicited by artworks, such as *anger*, *fear*, and *sadness* (Schindler et al., 2017); and epistemic and metacognitive theme-directed responses, such as *interest*, *reflection, awareness,* and *emotional connection,* to capture cognitive processing and reflective awareness and personal connection with the themes depicted (Morton, 2010; Vogl et al., 2021). Finally, an open-ended question asked participants to report any additional emotion evoked by the image.

*Familiarity* was assessed using a single item asking participants whether they were acquainted with the artwork, using a yes/no response format.

*Interest in street art* (ISA) was measured using an adaptation of the Graffiti/Street Art Interest Questionnaire (Gartus & Leder, 2014). The instrument has 10 items (e.g., “I am interested in street art”), which are answered on a 9-point scale (1 = “not at all” to 9 = “fully”). Reliability coefficients indicated good internal consistency (α =.84; ⍵ =.85); therefore, a global ISA was created with average scores (*M* = 7.29; *SD* = 1.26).

*Sustainability consciousness* was measured using the short version of the Sustainability Consciousness Questionnaire (SCQ-S; Gericke et al., 2018). To assess both attitudes and behaviors toward sustainability goals while reducing response burden, we focused on the Attitudes and Behavior dimensions, as the Knowledge items showed lower reliability in the original study (α =.70) and conceptual overlap with the Attitudes dimension. The scale included 18 items representing the *environmental* (e.g., “I recycle as much as I can”), *social* (“I support an aid organization or environmental group”), and *economic* (“I do things which help poor people”) pillars of Agenda 2030. Participants rated each statement on a scale from 1 (“strongly disagree”) to 5 (“strongly agree”). After evaluating reliability scores, we removed the inverted item (“I think that using more natural resources than we need does not threaten the health and well‐being of people in the future”) due to its low and inconsistent correlations (*r* ranging from −.17 to.07) with the other items. The final *sustainability consciousness* (SC) measure showed good reliability (α =.80, ⍵ =.84), allowing for a composite global SC measure based on average scores (*M* = 4.34; *SD* = 0.45).

*Attention check* was assessed by asking participants about their level of attention during their exposure and their responses following exposure to the images. Two response options were available: “No, I did not pay attention. I answered randomly to several questions” or “Yes, I paid attention” (e.g., Abbey & Meloy, 2017).

### Procedures

The project was approved by the University Ethical Committee (Ref. 62/2023) and conducted in accordance with ethical standards. After providing consent, participants completed an online survey via Qualtrics XM. A stimulus sampling design (SSD) was used, in which each participant rated 10 randomly selected images from a total of 556. Each image was displayed for at least 6 s, after which participants rated their emotions and familiarity. The survey also included sociodemographic questions, an attention check, the SCQ-S and ISA scales, an open comment section, and a debriefing. Due to anonymization procedures, it was not possible to verify multiple participations; however, the large image pool and randomization helped minimize this risk.

### Data analysis strategy

#### Descriptive statistics

We first conducted a descriptive analysis by calculating image-level averages for each emotion, providing a basic summary of the distribution of responses across images. Given our stimulus-sampling design, we then estimated adjusted predicted means for each emotion using cross-classified mixed-effects models (CC-MEM), with random intercepts for both participants and images (Huang & Jeon, 2022; Judd et al., 2019; Nestler & Back, 2017; Wickham et al., 2021), fitted with restricted maximum likelihood (REML). In these null models, we also computed the corresponding variance components and intraclass correlation coefficients (ICCs). Raw and model-adjusted image-level estimates were highly correlated across affective variables, ranging from *r* =.86 for awe to *r* =.99 for valence, indicating that the adjusted estimates preserved the overall descriptive pattern of the raw image-level means while accounting for the cross-classified structure of the data.

#### Dataset validation: Predictors of affective ratings

Next, we examined whether sociodemographic variables (nationality, gender, age), participants’ interest in street art (ISA), and sustainability consciousness (SC) contributed to explaining the variance in each affective response. All fixed effects were centered prior to analysis. We report the conditional *R*2 values (variance explained by the full model) and the marginal *R*^2^ (variance explained by the fixed effects). In addition, we provide the semi-partial *R*^2^ (i.e., the coefficient of partial determination, part *R*^2^), to quantify the proportion of the total variance uniquely attributable to each predictor while controlling for the remaining predictors. We also computed 95% confidence intervals (CIs) for several estimates. To compare model fit (null vs. full) for each outcome and to evaluate the incremental explanatory power of the fixed effects, we used the Akaike information criterion (AIC), the Bayesian information criterion (BIC), and the likelihood ratio test, fitting the linear multilevel models (MLMs) with maximum likelihood (ML). Lower AIC and BIC values indicate a better fit.

#### Dataset validation: Affective ratings interrelation

To investigate how affective responses covaried, we first examined how valence related to arousal and to specific affective labels. For all predictions involving valence, we tested both linear and quadratic terms. Model fit was compared using AIC, BIC, and likelihood-ratio tests. When analyzing how arousal related to affective responses, we restricted the analyses to linear models because they capture the intensity of the emotion. Across these analyses, each model included valence or arousal as the outcome variable, a single emotion label as the predictor, and random intercepts for both participants and images.

Given our large sample size, there was a likelihood of finding statistically significant results that were negligible or near zero. To address this concern across all linear MLMs, we applied the Anderson–Hauck (AH) test, a regression-based equivalence test that assesses whether predictor effects are negligible. Following recommendations that the smallest effect size of interest (SESOI) should reflect the natural variability of the construct (Anvari & Lakens, 2021; Lakens et al., 2018), we relied on the results from other normative affective datasets (e.g., OASIS, IAPS), which typically report SDs of about 1.0–1.7 for valence and arousal on 9-point scales (Kurdi et al., 2017). Thus, effects smaller than 20–30% of this variability can be considered trivial, and we therefore adopted a SESOI of ± 0.30 as a conservative threshold for defining negligible associations. Under this criterion, a significant AH test indicates that an effect has no practical impact on emotions, whereas non-significant AH results suggest that the effect may be meaningful. However, a quadratic coefficient is not directly comparable to a threshold defined in the linear regression, and published estimates for the valence-arousal quadratic term vary across visual affective datasets depending on stimulus set, rating methodology, and level of analysis (Kurdi et al., 2017; Marchewka et al., 2014), precluding the derivation of an empirically grounded SESOI. Therefore, for the quadratic models, we evaluated the overall *R*^2^ and also report the incremental variance explained compared to the linear models (Δ*R*2).

We then examined how the 11 rated emotion labels were interrelated through network analyses, rather than regression analyses, focusing on pairwise relationships. This approach also helps identify clusters of emotions that tend to co-occur and the bridges between them. For these analyses, we used participant-level adjusted means, previously estimated with the CC-MEM, to ensure that variability due to the random effects of images and participants was accounted for. A weighted network structure of partial correlation coefficients was estimated using the EBICglasso method, which integrates the extended Bayesian information criterion (Foygel & Drton, 2010) with the graphical LASSO (least absolute shrinkage and selection operator) using the recommended conservative parameter, γ = 0.5 (Epskamp & Fried, 2018). The resultant undirected network, part of a Gaussian graphical model (GGM), illustrates the connections between emotions by accounting for their interdependencies. For this estimation, we used the “signed” method to differentiate between positive and negative edges. Each node corresponds to an emotion, and the edge weights represent regularized partial correlation coefficients, of which the occurrence of minor or “spurious” edges will be diminished by shrinking them to zero, resulting in a network that predominantly features strong edges between two emotions, after controlling for all other variables in the network.

To examine the presence and structure of potential emotion clusters within the network, we also conducted an exploratory graph analysis (EGA) using the same regularized partial correlations with EBIC γ = 0.5, and the Louvain algorithm (Christensen et al., 2024). The accuracy and structural consistency of the findings were verified using bootstrap EGA with 1,000 bootstrap samples. Guidelines for item stability suggest that items with scores ≥.70 indicate good stability (minimal dimensional variability) (Christensen & Golino, 2021). Additionally, we examined the network loadings and estimated the roles of each emotion in connecting to different clusters using expected influence (EI) indexes, which consider both the strength and direction (positive or negative) of connections between emotions (Robinaugh et al., 2016). We calculated the “bridge expected influence (1-step)”, representing the sum of all connections between each emotion and all emotions in another cluster, and the “bridge expected influence (2-step)”, which also considers emotions that are indirectly related to the other emotions, where nodes with higher values indicate a greater bridging impact. Again, the accuracy and stability of these indices were assessed using 1,000 bootstrapped samples. Edge-weight accuracy was computed with 95% CIs via random resampling of the data. Correlation-stability (CS) coefficients evaluated replicability, measuring how much the sample size could be reduced while maintaining a correlation of at least.70 between the original and the bootstrapped samples. CS values above.50 indicate reliable interpretations (Epskamp et al., 2018). Comparisons between the original and bootstrapped samples were examined within 95% CIs to assess consistency.

Analyses were conducted using SPSS Statistics for Windows (version 29.0, IBM Corp.) and several packages from the R statistical environment (version 4.3.2; R Core Team, 2023), including *lme4* (v. 1.1–35.1, Bates et al., 2023) for the linear mixed models, *performance* (Lüdecke et al., 2021) for estimates of the ICCs, *partR2* (Stoffel et al., 2021) for marginal, conditional *R*^2^ and part *R*^2^, and *negligible* (Alter & Counsell, 2023) to assess whether fixed effects could be considered negligible. Network analyses were conducted using *networktools* (Jones, 2023), *EGAnet* (Golino & Christensen, 2023), and *bootnet* (Epskamp et al., 2018). Figures were created using *ggplot2* (Wickham, 2016), *magick* (Ooms, 2025), and *cowplot* (Wilke, 2024).

## Results

### Dataset creation: Overall description of the emotional responses toward the images

Each image was evaluated by 13 to 40 participants (*M* = 22). Most participants (78.7%, *n* = 975) were unfamiliar with the images they were exposed to. Table 2 presents descriptive statistics of affective responses to the street artworks based on aggregate values for the 556 images. The street art image stimuli (full-resolution and watermarked lower-resolution versions) and their corresponding metadata are available at https://doi.org/10.5281/zenodo.17702005. The metadata provide information for each image, including the artist’s name, artwork title, country, year of creation, and copyright holder. Access to the full-resolution images is granted upon approval of a Data Use Agreement (DUA), after which a password is provided.

**Table 2:** Descriptive statistics of affective responses to the 556 pictures of street art

	Min	Max	Range	*M*	*SD*	*SE*	*Med*	Skew	Kurtosis
Valence	1.62	8.47	6.86	5.53	1.44	0.06	5.75	−0.45	−0.59
Arousal	2.69	7.50	4.81	5.22	0.84	0.04	5.17	0.04	−0.25
Awe	2.73	6.11	3.38	4.43	0.67	0.03	4.39	0.01	−0.38
Moved	1.67	7.88	6.21	5.01	1.12	0.05	5.05	−0.01	−0.27
Inspired	1.39	7.17	5.78	4.39	1.14	0.05	4.40	<.001	−0.64
Hope	1.47	7.86	6.39	4.37	1.41	0.06	4.32	0.03	−0.96
Fear	1.07	7.12	6.05	3.16	1.31	0.06	2.90	0.72	−0.30
Sad	1.07	7.92	6.85	3.30	1.42	0.06	2.93	0.83	−0.08
Anger	1.05	7.70	6.65	2.92	1.41	0.06	2.47	1.11	0.56
Interest	2.85	7.41	4.56	5.52	0.72	0.03	5.55	−0.28	0.17
Reflect	2.87	8.12	5.25	5.73	0.96	0.04	5.73	−0.16	−0.30
Aware	2.60	8.23	5.63	5.55	1.04	0.04	5.53	−0.08	−0.45
Connect	2.07	7.58	5.51	5.15	1.00	0.04	5.16	−0.20	−0.44

Descriptive statistics were computed at the image level rather than at the individual participant level. Emotion labels are abbreviated: Moved = moved/touched; Hope = hopeful/optimistic; Awe = awe/amazement; Inspired = inspired/elevated; Interest = interested/curious; Fear = scared/afraid; Sad = sad/unhappy; Anger = angry/outraged; Connect = emotional connection to the theme; Reflect = reflection on the theme; Aware = awareness of the theme

The participant dataset and the R analysis code are available at https://doi.org/10.5281/zenodo.17966026. The shared materials include the original raw data in wide and long formats, raw image-level descriptive statistics, and model-adjusted image-level estimates derived from the multilevel null models. The raw descriptive file provides, for each image and emotion label, the number of ratings, mean, and standard deviation, both overall and by country/location, with separate values for Portuguese and Brazilian participants. The adjusted estimates were obtained from models including random intercepts for participants and images. An interactive website integrates the images with aggregated emotional ratings, enabling visual exploration of the artworks alongside their corresponding raw data for research purposes (https://www.patriciaarriaga.site/streetart4sustainability).

Overall, the artwork elicited mild pleasure (*valence*: *M* = 5.53; *SD* = 1.44; *Mdn* = 5.75) and moderate *arousal* (*M* = 5.22; *SD* = 0.84). The emotion labels with the highest scores were *reflection* (*M* = 5.73; *SD* = 0.96), *awareness* (*M* = 5.55; *SD* = 1.04), *interest* (*M* = 5.52; *SD* = 0.72), and *emotional connection* with the themes (*M* = 5.15; *SD* = 1.00), suggesting that the artwork elicited considerable cognitive engagement with the depicted themes. It also fostered being *moved* (*M* = 5.01; *SD* = 1.12), moderate *awe* (*M* = 4.43; *SD* = 0.67), *inspiration* (*M* = 4.39; *SD* = 1.14), and *hope* (*M* = 4.37; *SD* = 1.41). In contrast, the intensity of the negative emotions was lower (*M*_sadness_ = 3.30 ± 1.42; *M*_fear_ = 3.17 ± 1.31; *M*_anger_ = 2.92 ± 1.41). Some asymmetry was observed, but only in the distributions of valence (skew = − 0.45) and negative emotions (skew = 1.11, 0.83, and 0.72 for *anger*, *sadness*, and *fear*, respectively). The remaining emotions showed greater balance (skew between − 0.5 and 0.5).

**Fig. 1:**
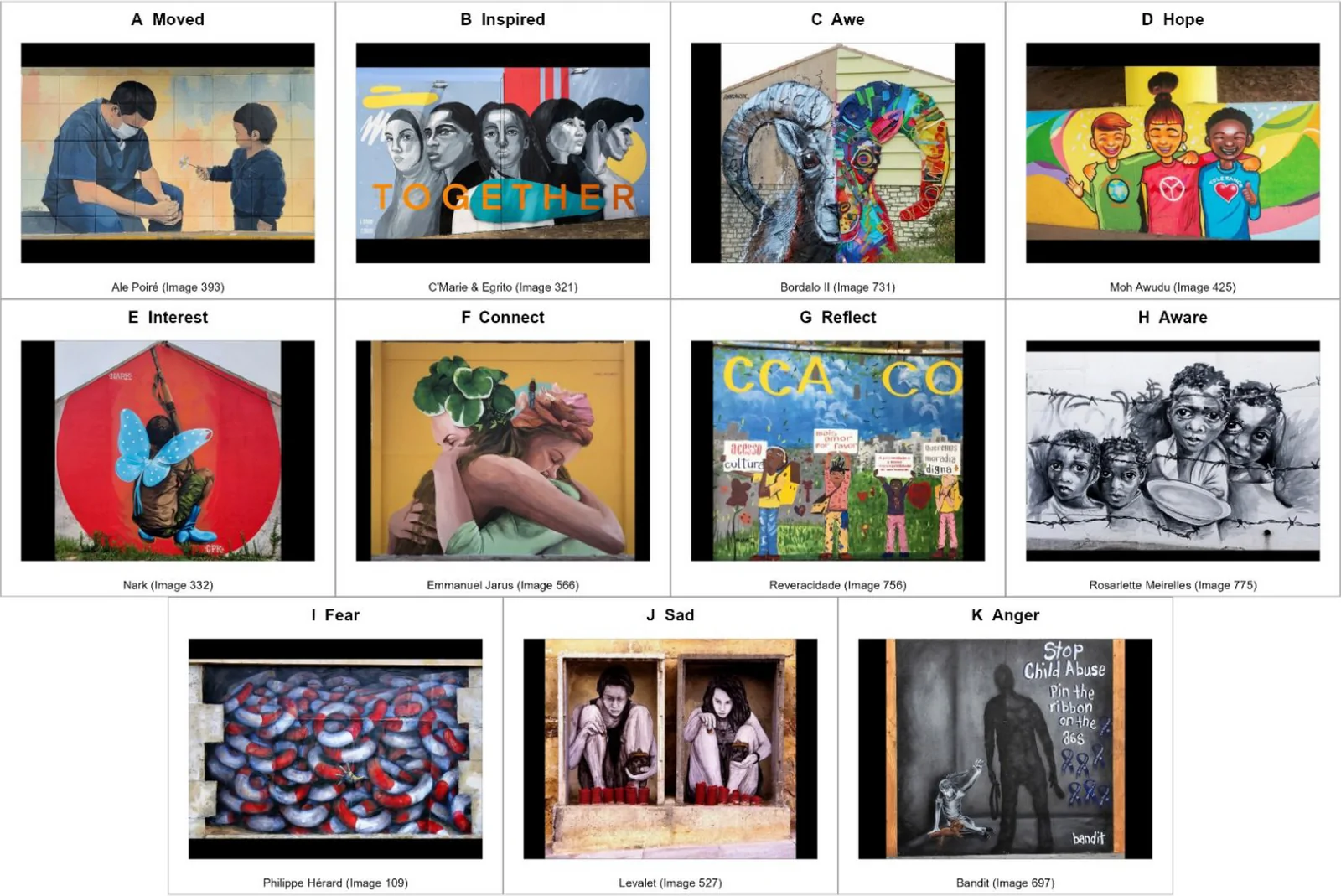
Example of artworks for each emotion label. Note. Images selected based on observed high mean ratings for the target emotion and a relatively higher mean compared with each of the other emotion labels. For each panel, the target emotion, artist/collective, and mean rating for the target emotion are presented: A = Moved, Ale Poiré, *M* = 7.57; B = Inspired, C'Marie & Egrito, *M* = 6.94; C = Awe, Bordalo II, *M* = 6.11; D = Hope, Moh Awudu, *M* = 7.86; E = Interest, Nark, *M* = 7.14; F = Connect, Emmanuel Jarus, *M* = 7.18; G = Reflect, Reveracidade, *M* = 7.82; H = Aware, Rosarlette Meirelles, *M* = 8.23; I = Fear, Philippe Hérard, *M* = 6.74; J = Sad, Levalet, *M* = 6.73; K = Anger, Bandit, *M* = 7.70

Figure 1 presents examples of the street artworks for each target emotion, together with the corresponding artist/collective, image identifier, and observed mean rating. The examples were selected based on their high mean ratings for the target emotion compared with other emotion labels, although each artwork elicited several affective responses.

### Dataset validation: Predictors of affective responses to the street artworks

Several MLMs were conducted to examine the variance explained in affective responses to the street artworks (see Table 3). Results from the null models showed high conditional *R*2 values for most affective responses, generally exceeding.40, with *hope* showing the highest (.49) and *arousal* the lowest (.25), though still relevant, highlighting the importance of including random intercepts for both participants and images. The ICCs related to the participant random effects were higher for positive emotions, ranging from.26 (*hope*) to.42 (*interest*). Conversely, the ICCs for participants were lower for *valence* (ICC =.13), *arousal*, and negative emotions (ICCs =.18), yet they still explained a relevant proportion of variance. For the random effect of images, ICCs ranged from.02 (*awe*) to.29 (*valence*) and were higher for *valence* and all *negative emotions* than for participants. In contrast, for all *positive emotions* and *arousal*, ICCs for participants consistently exceeded those of images.

**Table 3:** Multilevel linear regression models for affective responses with demographics, interest in street art, and sustainability consciousness as predictors

Model Statistics	Null	Full	Null	Full	Null	Full	Null	Full	Null	Full
	**Valence**		**Arousal**		**Moved**		**Awe**		**Inspired**	
(Intercept) *b₀* [95% CI]	5.53^***^ [5.40, 5.66]	5.53^***^ [5.41, 5.66]	5.23^***^ [5.14, 5.32]	5.20^***^ [5.11, 5.29]	5.04^***^ [4.92, 5.16]	4.99^***^ [4.88, 5.11]	4.47^***^ [4.37, 4.57]	4.47^***^ [4.37, 4.56]	4.40^***^ [4.28, 4.53]	4.39^***^ [4.27, 4.51]
*SE*	0.07	0.07	0.05	0.05	0.06	0.06	0.05	0.05	0.06	0.06
Fixed										
Nationality* b* [95%CI]		− 0.07^*^ [− 0.13, − 0.01]		− 0.14^***^ [− 0.21, − 0.07]		− 0.35^***^ [− 0.44, − 0.26]		− 0.34^***^ [− 0.43, − 0.24]		− 0.17^***^ [− 0.25, − 0.08]
*SE*		0.03		0.04		0.04		0.05		0.04
Part *R*^2^ [95%CI]		.001 [0,.01]		.003 [.001,.01]		.02 [.01,.04]		.02 [.001,.02]		.004 [.001, 0.01]
AH		−2.30^***^		3.83^***^		−7.93		−6.98		−3.93^***^
Gender *b* [95% CI]		0.07^*^ [0.01, 0.13]		− 0.07 [− 0.14,0.001]		− 0.04 [− 0.13, 0.05]		0.18^***^ [0.09, 0.28]		0.12^**^ [0.04, 0.21]
*SE*		0.03		0.04		0.04		0.05		0.04
Part *R*^2^ [95% CI]		.001 [.0001,.008]		.001 [0,.01]		.0002 [0,.02]		.005 [0,.01]		.002 [.0002,.012]
AH		2.43^***^		−1.94^***^		0.91^***^		3.73^***^		2.93^***^
Age *b* [95% CI]		− 0.004 [− 0.01,0.002]		0.002 [− 0.004,0.01]		0.01^***^ [0.01, 0.02]		− 0.01 [− 0.02, 0.000]		0.01^*^ [0.001, 0.02]
*SE*		0.003		0.003		0.004		0.004		0.004
Part *R*^2^ [95% CI]		.0003 [0,.01]		.0001 [0,.01]		0.003 [0,.02]		0.001 [0, 0.01]		.001 [0,.01]
AH		−1.33^***^		0.67^***^		3.25^***^		−1.75^***^		2.00^***^
ISA *b* [95% CI]		0.32^***^ [0.27, 0.37]		0.11^***^ [0.05, 0.17]		0.41^***^ [0.34, 0.49]		0.41^***^ [0.32, 0.49]		0.40^***^ [0.33, 0.47]
*SE*		0.03		0.03		0.04		0.04		0.04
Part *R*^2^ [95% CI]		.02 [.02,.03]		.003 [.001,.01]		.03 [.02,.05]		.03 [.02,.04]		.03 [.02, 0.04]
AH		11.81		3.65^***^		10.89		9.64		10.78
SC *b* [95% CI]		0.16^*^ [0.01, 0.31]		0.28^**^ [0.10, 0.46]		0.56^***^ [0.34, 0.78]		0.22 [− 0.02, 0.46]		0.53^***^ [0.32, 0.74]
*SE*		0.08		0.09		0.11		0.12		0.11
Part *R*^2^ [95% CI]		.001 [0,.01]		.002 [.0001,.01]		.01 [0, 0.03]		.001 [0, 0.01]		.006 [.001,.02]
AH		2.04^***^		3.08		5.04		1.80		4.93
Random										
Participant										
τ^2^ (*SD* τ)	0.87 (0.93)	0.67 (0.82)	1.10 (1.05)	1.01 (1.01)	2.46 (1.57)	1.78 (1.33)	2.75 (1.66)	2.25 (1.50)	2.07 (1.44)	1.59 (1.26)
ICC	.13	.11	.18	.17	.34	.27	.41	.36	.28	.23
Picture										
τ^2^ (*SD* τ)	1.87 (1.37)	1.87 (1.37)	0.44 (0.66)	0.44 (0.66)	0.90 (0.95)	0.90 (0.95)	0.11 (0.34)	0.11 (0.34)	1.02 (1.01)	1.02 (1.01)
ICC	.29	.30	.07	.07	.13	.14	.02	.02	.14	.15
σ^2^ (σ)	3.81 (1.95)	3.81 (1.95)	4.56 (2.13)	4.55 (2.13)	3.83 (1.96)	3.83 (1.96)	3.89 (1.97)	3.89 (1.97)	4.21 (2.05)	4.20 (2.05)
*R*^2^_Conditional_	.42	.42	.25	.25	.47	.47	.42	.43	.42	.42
*R*^2^_Marginal_	–	.03	–	.01	–	.09	–	.08	–	.07
AIC	51196	51028	52610	52579	51892	51601	51467	51289	52707	52484
BIC	51226	51094	52640	52645	51922	51667	51496	51355	52737	52550
χ2 (5)	207.60^***^		69.29^***^		327.82^***^		213.36^***^		259.91^***^	
	**Hope**		**Reflect**		**Aware**		**Connect**		**Interest**	
(Intercept) *b₀* [95% CI]	4.39^***^ [4.25, 4.52]	4.36^***^ [4.23, 4.50]	5.75^***^ [5.63, 5.87]	5.71^***^ [5.60, 5.81]	5.57^***^ [5.45, 5.69]	5.53^***^ [5.41, 5.64]	5.17^***^ [5.06, 5.29]	5.15^***^ [5.04, 5.25]	5.55^***^ [5.44, 5.65]	5.52^***^ [5.42, 5.61]
*SE*	0.07	0.07	0.06	0.06	0.06	0.06	0.06	0.06	0.05	0.05
Fixed										
Country* b* [95% CI]		− 0.23^***^ [− 0.31, − 0.15]		− 0.25 ^***^ [− 0.33, − 0.16]		− 0.20^***^ [− 0.29, − 0.11]		− 0.14^**^ [− 0.23, − 0.05]		− 0.21^***^ [− 0.30, − 0.12]
*SE*		0.04		0.05		0.05		0.04		0.05
Part *R*^2^ [95% CI]		.01 [0, 0.018]		.01 [.001, 0.02]		.01 [.001,.02]		.002 [0,.01]		.01 [0, 0.03]
AH		5.83^***^		−5.49^***^		−4.41^***^		−3.14^***^		−4.61^***^
Gender *b* [95% CI]		0.07 [− 0.01, 0.15]		− 0.06 [− 0.15, 0.03]		− 0.08 [− 0.17, 0.01]		− 0.004 [− 0.09, 0.08]		0.02 [− 0.07, 0.11]
*SE*		0.04		0.05		0.05		0.05		0.05
Part *R*^2^ [95% CI]		.001 [0, 0.01]		.0004 [0,.02]		.001 [0,.02]		<.001 [0, 0.01]		<.001 [0,.02]
AH		1.80^***^		−1.27^***^		−1.72^***^		−0.09^***^		0.39^***^
Age *b* [95% CI]		0.01^***^ [0.01, 0.018]		− 0.002 [− 0.01, 0.01]		0.003 [− 0.004, 0.01]		0.01^**^ [0.003, 0.02]		− 0.005 [− 0.01, 0.003]
*SE*		0.003		0.004		0.004		0.004		0.004
Part *R*^2^ [95% CI]		.002 [0,.01]		.0001 [0,.02]		.0002 [0,.02]		.002 [0,.01]		.001 [0,.02]
AH		3.67^***^		−0.50^***^		0.75^***^		2.50^***^		−1.25^***^
ISA *b* [95% CI]		0.40^***^ [0.33, 0.47]		0.53^***^ [0.46, 0.61]		0.53^***^ [0.45, 0.60]		0.51^***^ [0.43, 0.59]		0.54^***^ [0.46, 0.62]
*SE*		0.04		0.04		0.04		0.04		0.04
Part*R*^2^ [95% CI]		.03 [.02,.04]		.05 [.04,.07]		.05 [.03,.07]		.04 [.04,.05]		.06 [.04,.07]
AH		11.43		14.05		13.15		13.42		13.50
SC *b* [95% CI]		0.45^***^ [0.25, 0.65]		0.64^***^ [0.42, 0.86]		0.62^***^ [0.40, 0.85]		0.56^***^ [0.34, 0.78]		0.51^***^ [0.28, 0.74]
*SE*		0.10		0.11		0.12		0.11		0.12
Part*R*^2^ [95% CI]		.004 [0,.02]		.01[.001,.03]		.01[.001,.03]		.01 [.001,.02]		.01 [0,.02]
AH		4.42		5.72		5.37		5.03		4.39
Random										
Participant										
τ^2^ (*SD* τ)	1.93 (1.39)	1.44 (1.20)	2.67 (1.63)	1.86 (1.36)	2.75 (1.66)	1.99 (1.41)	2.45 (1.56)	1.77 (1.33)	2.73 (1.65)	2.02 (1.42)
ICC	.26	.21	.39	.30	.38	.30	.33	.26	.42	.34
Picture										
τ^2^ (*SD* τ)	1.71 (1.31)	1.71 (1.31)	0.60 (0.77)	0.60 (0.77)	0.74 (0.86)	0.74 (0.86)	0.64 (0.80)	0.64 (0.80)	0.20 (0.44)	0.20 (0.44)
ICC	.23	.25	.09	.10	.10	.11	.09	.10	.03	.03
σ^2^ (σ)	3.85 (1.96)	3.85 (1.96)	3.67 (1.91)	3.66 (1.91)	3.81 (1.95)	3.81 (1.95)	4.38 (2.09)	4.38 (2.09)	3.65 (1.91)	3.65 (1.91)
*R*^2^_Conditional_	.49	.49	.47	.47	.48	.48	.41	.41	.45	.45
*R*^2^_Marginal_	–	.07	–	.12	–	.11	–	.09	–	.11
AIC	51990	51732	51335	50993	51866	51561	53109	52822	50935	50649
BIC	52020	51799	51365	51060	51896	51627	53138	52888	50965	50715
χ2 (5)	294.61^***^		377.56^***^		341.07^***^		322.90^***^		322.31^***^	
	**Sad**		**Fear**		**Anger**					
(Intercept) *b₀* [95% CI]	3.31^***^ [3.18, 3.44]	3.29^***^ [3.16, 3.43]	3.17^***^ [3.05, 3.30]	3.16^***^ [3.03, 3.28]	2.92^***^ [2.79, 3.05]	2.92^***^ [2.79, 3.05]				
*SE*	0.07	0.07	0.06	0.06	0.07	0.07				
Fixed										
Nationality* b* [95% CI]		− 0.03 [− 0.10, 0.05]		− 0.12** [− 0.19, − 0.04]		0.07 [− 0.003,0.14]				
*SE*		0.04		0.04		0.04				
Part *R*^2^ [95% CI]		.0001 [0,.01]		.002 [.001,.01]		.001 [.0001,.005]				
AH		−0.74^***^		−3.16^***^		1.86^***^				
Gender *b* [95% CI]		− 0.08^*^ [− 0.15, − 0.003]		− 0.07 [− 0.14, 0.01]		− 0.02 [− 0.09, 0.05]				
*SE*		0.04		0.04		0.04				
Part *R*^2^ [95% CI]		.001 [.0001,.01]		.001 [0,.004]		.0001 [0,.004]				
AH		−2.05^***^		1.81^***^		−0.54^***^				
Age *b* [95% CI]		< 0.001 [− 0.01, 0.01]		0.002 [− 0.01, 0.01]		0.004 [− 0.002,0.01]				
*SE*		0.003		0.003		0.003				
Part *R*^2^ [95% CI]		< 0.001 [0,.01]		0 [0,.003]		.0003 [0,.004]				
AH		0.03^***^		0.67^***^		1.33^***^				
ISA *b* [95% CI]		0.02 [− 0.04, 0.09]		0.01 [− 0.05, 0.08]		− 0.01 [− 0.01, 0.06]				
*SE*		0.033		0.03		0.03				
Part *R*^2^ [95% CI]		.0001 [0,.006]		< 0.001 [0,.003]		< 0.001 [0,.004]				
AH		0.73^***^		0.41^***^		−0.19^***^				
SC *b* [95% CI]		0.20* [0.01, 0.39]		0.10 [− 0.01, 0.28]		0.11 [− 0.07, 0.29]				
*SE*		0.10		0.09		0.09				
Part *R*^2^ [95% CI]		.001 [.0001,.001]		.0002 [0,.003]		.0003 [0,.004]				
AH		2.05		1.03^***^		1.17^***^				
Random										
Participant										
τ^2^ (*SD* τ)	1.26 (1.12)	1.25 (1.12)	1.19 (1.09)	1.18 (1.09)	1.14 (1.07)	1.14 (1.07)				
ICC	.18	.18	.18	.18	.18	.18				
Picture										
τ^2^ (*SD* τ)	1.75 (1.32)	1.75 (1.32)	1.48 (1.22)	1.48 (1.22)	1.77 (1.33)	1.77 (1.33)				
ICC	.26	.26	.23	.23	.27	.27				
σ^2^ (σ)	3.85 (1.96)	3.85 (1.96)	3.81 (1.95)	3.807 (1.95)	3.56 (1.89)	3.56 (1.89)				
*R*^2^_Conditional_	.44	.44	.41	.41	.45	.45				
*R*^2^_Marginal_	–	.003	–	.003	–	.001				
AIC	51592	51615	51353	51374	50720	50750				
BIC	51622	51681	51383	51440	50749	50816				
χ2 (5)	14.72*		16.82**		7.57					

^*^p <.05, ^**^p <.01, ^***^p <.001. *N*_Observations_ = 11,614; *N*_Pictures_ = 556; *N*_Participants_ = 1,217; The fixed variables are mean-centered; Nationality (−1 = Brazilian; 1 = Portuguese); Gender (−1 = Women; 1 = Men); ISA = Interest in Street Art; SC = Sustainability Consciousness; CI = Confidence Interval; AIC = Akaike Information Criterion; BIC = Bayesian information criterion; ML = maximum likelihood; *R*^2^_Conditional_ = Variance explained by both fixed and random effects; *R*^2^_Marginal_ = Variance Explained by Fixed Effects; Part *R*^2^ = unique contributions of each predictor; AH = Anderson-Hauck T statistic; Model comparisons with ANOVAs were calculated using Maximum Likelihood (ML), for all the other estimates Restricted Maximum Likelihood (REML) was used. Affective labels are abbreviated: Moved = moved/touched; Hope = hopeful/optimistic; Awe = awe/amazement; Inspired = inspired/elevated; Interest = interested/curious; Fear = scared/afraid; Sad = sad/unhappy; Anger = angry/outraged; Connect = emotional connection to the theme; Reflect = reflection on the theme; Aware = awareness of the theme

The full model incorporated five fixed effects (nationality, gender, age, ISA, and SC) and the random intercepts for participants and images to predict emotional responses. Adding the fixed variables resulted in a statistically significant, albeit modest, increase in explained variance for *valence*, *arousal*, and all *positive emotions* (*p*s <.001). Additionally, decreases in ΔAIC and ΔBIC values indicated that the full models achieved a better balance between model complexity and fit. Regarding *valence*, the marginal *R*2 was 3.10%. However, despite the statistically significant effects of nationality, ISA, SC, and gender, the results of the AH *t*-test indicated that most of the variance was uniquely explained by ISA, with the remaining variance accounted for by the other variables considered negligible (part *R*^2^ ≤ 0.1%). For *arousal*, the fixed effects collectively explained 1.4% of the variance. However, except for SC, which had a small unique contribution (part *R*^2^ = 0.2%), the effects of the other variables were also considered negligible based on the AH *t*-test. For the specific *positive emotions*, ISA and SC were significant predictors of *reflect* (marginal *R*2 = 12%), *interest* (marginal *R*2 = 11%), *aware* (marginal *R*2 = 11%), *connect* (marginal *R*2 = 9%), *inspired*, and *hope* (both with marginal *R*2 = 7%). In the case of *moved* (marginal *R*2 = 8%), ISA, SC, and nationality all showed non-negligible unique contributions (part *R* 2 s ≈.01–.03). For *awe* (marginal *R*2 = 8%), only ISA and nationality were predictors with non-negligible effects. For the negative emotions, adding fixed predictors did not improve models’ performance, as reflected by positive ΔBIC values for the three negative emotions and a nonsignificant analysis of variance (ANOVA) for *anger*. Only SC on sadness was non-negligible by the AH test (b = 0.20, part *R*² = .001). Marginal *R*^2^ values were also very low (<.004 for negative emotions), indicating that the predictors collectively contributed little to explaining the variance in these emotions.

Overall, ISA had the highest partial *R*2 values, uniquely explaining more variance in most positive emotions than other predictors, with its largest contribution being a 5.5% explanation of variance in reported interest in the artworks. SC also contributed to explaining variance in most positive emotions and in *arousal*, albeit to a lesser extent. Among the remaining predictors, only nationality made a modest contribution to predicting feelings of *moved* and *awe*, with Brazilian participants reporting higher intensities of these emotions. In contrast, the effects of gender and age were negligible, indicating that women and men reported similar affective experiences and that these affective responses were consistent across age groups. Overall, these findings suggest that ISA and SC play a more prominent role in shaping affective experiences toward artworks in our database than the demographic variables considered.

### Dataset validation: Interrelationship between affective responses

To examine whether *valence* and specific emotional responses varied either linearly or nonlinearly across the valence continuum, we estimated MLMs predicting each affective response from linear and quadratic valence terms.

The associations between *valence*, *arousal*, and each emotional response are summarized in Table 4. In the linear models, *valence* was statistically significantly associated with all outcomes. However, the AH tests indicated that only five of these linear associations were non-negligible, exceeding the predefined SESOI threshold: *inspired* (*b* = 0.53) and *hope* (*b* = 0.58) in the positive direction, and *sadness* (*b* = − 0.35), *fear* (*b* = − 0.36), and *anger* (*b* = − 0.36) in the negative direction. The quadratic regression models varied across outcomes, suggesting three main patterns. First, for *inspired* and *hope*, the linear models already accounted for a substantial proportion of variance (marginal *R*2 =.27 and.33, respectively), and the quadratic terms produced only modest additional increases in explained variance (marginal Δ*R*2 =.05 and.04, respectively). These responses are therefore best described as showing predominantly positive linear associations with *valence*, with some additional curvature. Second, for *sadness*, *fear*, and *anger*, the negative linear associations were also non-negligible, whereas the quadratic terms added little explanatory value (marginal Δ*R*2 ≤.01), providing limited evidence for curvilinear patterns. Third, for *connection*, *moved*, *interest*, *reflection*, *awareness*, and *awe*, the linear associations with *valence* were classified as negligible by the AH tests, whereas the quadratic models improved model fit, with explained variance varying across outcomes (quadratic marginal *R*2 =.05 to.13; marginal Δ*R*2 =.03 to.07). This pattern suggests modest curvilinear patterns, with stronger responses at more polarized levels of valence than at intermediate levels. *Arousal* showed a similar but weaker pattern (quadratic marginal *R*2 =.02; marginal Δ*R*2 =.02).

**Table 4:** Multilevel linear and quadratic regression models for valence as predictor with arousal and specific emotional labels as outcomes

Model	Outcome	*B* [95%CI]	*SE*	*R*^2^_Marginal_	Δ*R*^2^	ICC_Participant_	ICC_Picture_	AH	AIC	BIC	χ^2^(1)
Linear	Arousal	−0.05^***^ [−0.07, −0.03]	0.009	.003	–	.19	.07	5.08^***^	53,290	53,327	
Quadratic	Arousal	0.05^***^ [0.05, 0.06]	0.004	.02	.02	.18	.06	–	53,079	53,123	213.43^***^
Linear	Moved	0.20^***^ [0.18, 0.22]	0.009	.04	–	.31	.15	22.03^***^	52,160	52,196	
Quadratic	Moved	0.10^***^ [0.10, 0.11]	0.003	.10	.06	.30	.14	–	51,257	51,301	904.95^***^
Linear	Awe	0.15^***^ [0.13, 0.17]	0.009	.02	–	.39	.02	17.54^***^	51,923	51,960	
Quadratic	Awe	0.06^***^ [0.06, 0.07]	0.003	.05	.03	.38	.02	–	51,590	51,635	334.91^***^
Linear	Inspired	0.53^***^ [0.51, 0.54]	0.008	.27	–	.29	.04	64.90	50,170	50,207	
Quadratic	Inspired	0.08^***^ [0.08, 0.09]	0.003	.32	.05	.27	.04	–	49,497	49,542	674.55^***^
Linear	Hope	0.58^***^ [0.56, 0.60]	0.008	.33	–	.28	.10	74.45	48,461	48,498	
Quadratic	Hope	0.08^***^ [0.07, 0.08]	0.003	.37	.04	.26	.09	–	47,830	47,874	633.38^***^
Linear	Reflect	0.16^***^ [0.15, 0.18]	0.009	.03	–	.36	.11	18.33^***^	51,754	51,791	
Quadratic	Reflect	0.09^***^ [0.08, 0.09]	0.003	.07	.04	.35	.10	–	51,127	51,171	628.92^***^
Linear	Aware	0.14^***^ [0.12, 0.16]	0.009	.02	–	.36	.12	15.00^***^	52,410	52,447	
Quadratic	Aware	0.09^***^ [0.08, 0.10]	0.003	.06	.04	.34	.11	–	51,778	51,823	633.23^***^
Linear	Connect	0.24^***^ [0.22, 0.26]	0.010	.05	–	.30	.10	25.11^***^	53,265	53,302	
Quadratic	Connect	0.11^***^ [0.11, 0.12]	0.004	.13	.07	.29	.08	–	52,286	52,330	981.00^***^
Linear	Interest	0.25^***^ [0.24, 0.27]	0.008	.07	–	.40	.03	30.86^***^	50,761	50,798	
Quadratic	Interest	0.07^***^ [0.06, 0.08]	0.003	.10	.03	.38	.03	–	50,325	50,369	438.73^***^
Linear	Sad	−0.35^***^ [−0.37, −0.33]	0.009	.13	–	.24	.15	40.03	50,916	50,953	
Quadratic	Sad	0.02^***^ [0.02, 0.03]	0.003	.13	.01	.24	.14	–	50,866	50,910	51.76^***^
Linear	Fear	−0.36^***^ [−0.38, −0.34]	0.009	.14	–	.25	.11	42.06	50,582	50,619	
Quadratic	Fear	0.02^***^ [0.01, 0.02]	0.003	.14	.003	.24	.11	–	50,556	50,601	27.72^***^
Linear	Anger	−0.36^***^ [−0.37, −0.34]	0.008	.14	–	.24	.16	42.47	49,852	49,889	
Quadratic	Anger	0.03^***^ [0.03, 0.04]	0.003	.15	.01	.23	.16	–	49,746	49,790	108.21^***^

^***^p <.001. *N*_Observations_ = 11,779; *N*_Pictures_ = 556; *N*_Participants_ = 1,234; CI = Confidence Interval; AIC = Akaike Information Criterion; BIC = Bayesian information criterion; The fixed variables are mean-centred; *R*^2^_Marginal_ = Variance explained by fixed effects; AH = Anderson-Hauck T statistic (result considered negligible if its estimated coefficient falls within the predefined Smallest Effect Size of Interest (SESOI) of ± 0.30, with *p* <.05). Model Comparisons with ANOVAs were calculated using maximum likelihood (ML), for all the other estimates restricted maximum likelihood (REML) was used. Emotion labels are abbreviated: Moved = moved/touched; Hope = hopeful/optimistic; Awe = awe/amazement; Inspired = inspired/elevated; Interest = interested/curious; Fear = scared/afraid; Sad = sad/unhappy; Anger = angry/outraged; Connect = emotional connection to the theme; Reflect = reflection on the theme; Aware = awareness of the theme

Across all linear models in which *arousal* predicted affective responses, despite statistically significant results, the AH tests using the predefined SESOI indicated that all effects were negligible (see Table 5).

**Table 5:** Multilevel linear regression models for arousal as predictor with valence and specific emotional labels as outcomes

Predictor	*b* [95% CI]	*SE*	*R*^2^_Marginal_	ICC_Participant_	ICC_Picture_	AH
Valence	−0.01 [−0.03, 0.004]	0.009	<.001	.13	.28	33.48^***^
Moved	0.18^***^ [0.16, 0.20]	0.009	.03	.32	.13	13.74^***^
Awe	0.16^***^ [0.14, 0.18]	0.009	.02	.39	.01	16.66^***^
Inspired	0.08^***^ [0.06, 0.10]	0.009	.005	.27	.15	24.28^***^
Hope	0.04^***^ [0.02, 0.06]	0.009	.001	.25	.24	29.21^***^
Reflect	0.17^***^ [0.16, 0.19]	0.009	.03	.37	.09	15.07^***^
Aware	0.19^***^ [0.17, 0.21]	0.009	.03	.36	.10	12.85^***^
Connect	0.18^***^ [0.16, 0.20]	0.009	.03	.31	.09	13.10^***^
Interest	0.14^***^ [0.12, 0.16]	0.008	.02	.40	.03	19.18^***^
Sad	0.17^***^ [0.15, 0.18]	0.009	.03	.18	.25	15.55^***^
Fear	0.19^***^ [0.17, 0.20]	0.008	.03	.18	.21	13.46^***^
Anger	0.18^***^ [0.17, 0.20]	0.008	.03	.18	.26	14.23^***^

^***^p <.001. *N*_Observations_ = 11,779; *N*_Pictures_ = 556; *N*_Participants_ = 1234; The fixed variables are mean-centered; *R*^2^_Marginal_ = Variance explained by fixed effects; AH = Anderson-Hauck T statistic (result considered negligible if its estimated coefficient falls within the predefined Smallest Effect Size of Interest (SESOI) of ± 0.30. Emotion labels are abbreviated: Moved = moved/touched; Hope = hopeful/optimistic; Awe = awe/amazement; Inspired = inspired/elevated; Interest = interested/curious; Fear = scared/afraid; Sad = sad/unhappy; Anger = angry/outraged; Connect = emotional connection to the theme; Reflect = reflection on the theme; Aware = awareness of the theme

Finally, to analyze the structure of specific emotion labels, we first examined the redundancy among these emotions before estimating the network. Using the *goldbricker* function (Jones, 2023) (*p* =.05, *corMin* =.50, difference threshold of.20), no pairs of emotions were identified as redundant, indicating that all emotions were suitable for inclusion in the network model. The estimated network density of all 11 emotion ratings, based on partial correlations (EBICglasso), was 72.73%, reflecting 40 out of 55 possible connections (sparsity =.27 and *M*_edge weight_ =.09). Table 6 shows the weighted adjacency matrix of the network structure, revealing strong associations among several emotions and suggesting the need for further investigation into how these emotions may be organized into distinct clusters.

**Table 6:** Weighted adjacency matrix with partial correlations among the specific emotional labels

	Moved	Hope	Awe	Inspired	Interest	Connect	Reflect	Aware	Fear	Sad
Hope	.371	–								
Awe	.123	.033	–							
Inspired	0	.567	.217	–						
Interest	.037	0	.250	.102	–					
Connect	.255	.015	0	.197	.023	–				
Reflect	.033	0	-.025	0	.358	.174	–			
Aware	.071	.006	-.027	0	.037	.188	.696	–		
Fear	.027	0	.112	-.018	-.002	0	0	.009	–	
Sad	.141	-.112	0	-.025	0	0	.030	0	.427	–
Anger	0	-.069	0	.016	-.038	.089	-.005	.016	.440	.403

Values represent EBICglasso-regularized partial correlations among emotional labels. They indicate conditional associations between pairs of labels after accounting for the remaining labels in the network. Emotional labels are abbreviated: Moved = moved/touched; Hope = hopeful/optimistic; Awe = awe/amazement; Inspired = inspired/elevated; Interest = interested/curious; Fear = scared/afraid; Sad = sad/unhappy; Anger = angry/outraged; Connect = emotional connection to the theme; Reflect = reflection on the theme; Aware = awareness of the theme

**Fig. 2:**
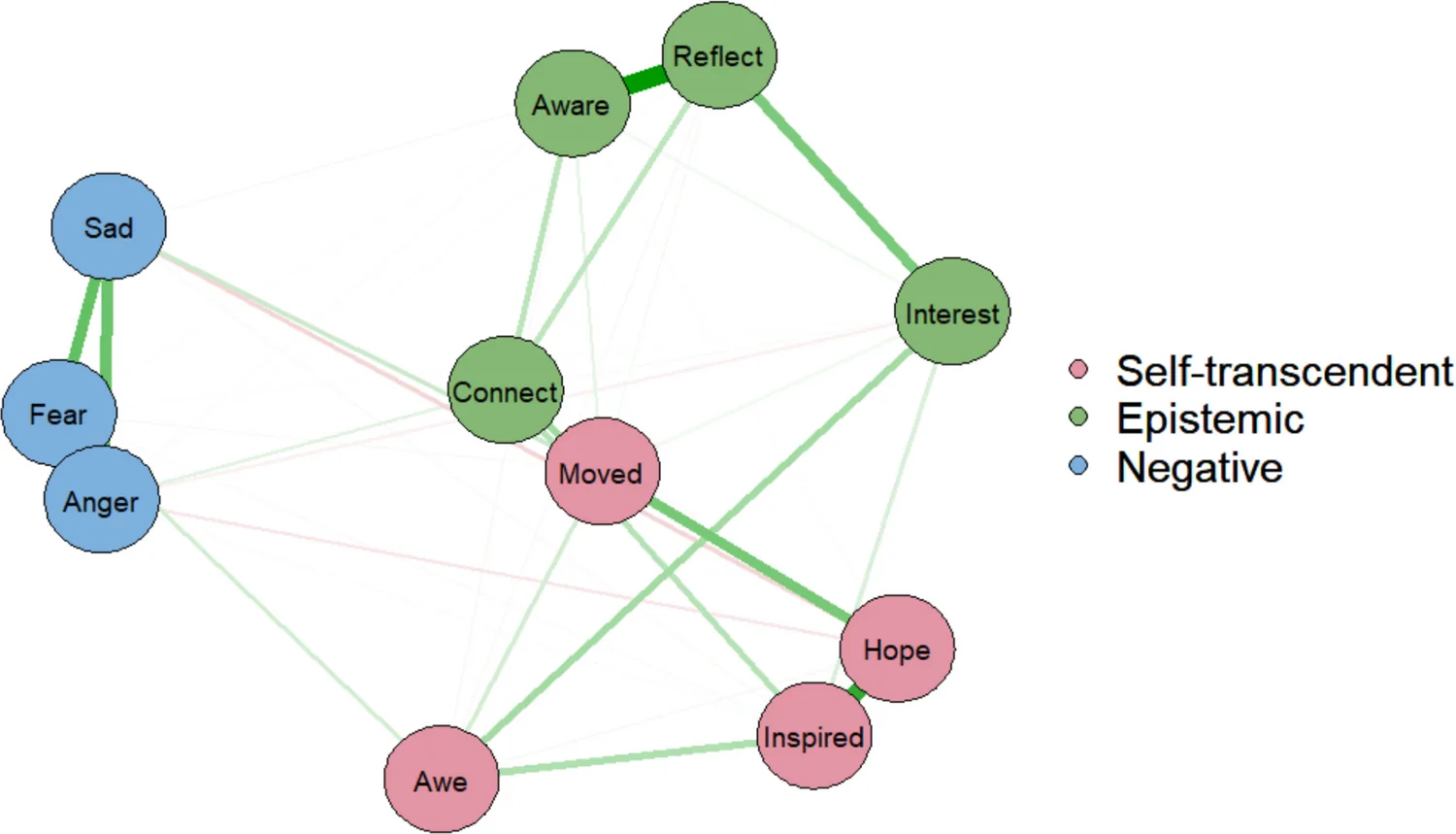
Emotional network structure of the affective labels. Note. Exploratory Graph Analysis using multidimensional scaling (MDSnet); Nodes = emotion labels; Edges = Regularized partial correlations (with LASSO penalty; Louvain algorithm). Distance between nodes and thickness of edges = strength of associations: green (positive), red (negative). Dimensions: *Self-transcendent* (pink), *Epistemic* (green), and *Negative* (blue). Emotion labels are abbreviated: *Moved* = moved/touched; *Hope* = hopeful/optimistic; *Awe* = awe/amazement; *Inspired* = inspired/elevated; *Interest* = interested/curious; *Fear* = scared/afraid; *Sad* = sad/unhappy; *Anger* = angry/outraged; *Connect* = emotional connection to the theme; *Reflect* = reflection on the theme; *Aware* = awareness of the theme

Using EGA with the Louvain algorithm, we identified three dimensions. The network exhibited moderate overall connectedness (edge density =.62). Edge weights were, on average, moderate (*M* =.15, *SD* =.15), with most variables positively related. The first dimension (D1) was named *self-transcendent,* comprising the emotions *moved*, *awe*, *inspired*, and *hope*. The second dimension (D2) includes *interest*, *reflection*, *awareness*, and *connection* with the theme portrayed. Although broader than epistemic emotion in a strict sense, we label this dimension *epistemic* because it captures curiosity, meaning-making, and reflective engagement with the issues depicted. Finally, the third dimension (D3), designated as *negative emotions*, includes *anger*, *fear*, and *sadness*. Applying bootstrapping with 1,000 replications identified the same three dimensions in 909 instances, compared to only 91 occurrences for a four-dimensional structure, as indicated by the median [2.44, 3.56] 95% CI. Both network models (one based on the empirical dataset and the other on 1,000 bootstrap samples) yielded similar results. Figure 2 presents the emotional network using multidimensional scaling. To complement these results, we examined the network loadings to assess the degree of association between each emotion and its respective dimension (see Table 7). Standardized loadings with absolute values ≥.20 were considered salient and supported the three-dimensional structure (loadings from.29 to.63 for *self-transcendent*; from.33 to.72 for *epistemic*; and from.59 to.60 for *negative emotions*). Nevertheless, *moved*, *interest*, and *connect* also displayed cross-loadings above |.20| on *self-transcendent* and *epistemic* dimensions, reflecting close relations between these response domains.

**Table 7:** Network loadings for the three dimensions of specific emotional labels

Emotions	Self-Transcendent	Epistemic	Negative
Hope	.625	.033	−.010
Inspired	.554	.159	0
Moved	.351	.242	.038
Awe	.294	.110	.035
Reflect	.046	.719	.009
Aware	.063	.612	.013
Interest	.223	.343	0
Connect	.261	.329	.019
Fear	.057	.011	.596
Anger	−.002	.020	.588
Sad	.019	.013	.586

Emotion labels are abbreviated: Moved = moved/touched; Hope = hopeful/optimistic; Awe = awe/amazement; Inspired = inspired/elevated; Interest = interested/curious; Fear = scared/afraid; Sad = sad/unhappy; Anger = angry/outraged; Connect = emotional connection to the theme; Reflect = reflection on the theme; Aware = awareness of the theme

The structural consistency values were high (.99 for D1,.93 for D2, and 1.00 for D3), indicating strong internal consistency among emotions within each dimension. Replication rates for emotion labels within their respective dimensions were also high (94% for *connect* (D2); 99% for *interest* (D2) and *moved* (D1); and 100% for the remaining eight emotion labels). The model with 1,000 bootstrapped networks also showed high stability for the bridge’s expected influence (BEI), with a correlation-stability (CS) coefficient of.75, indicating that the correlation between the original and bootstrapped samples remained strong, even with subsets comprising 30% of the original sample (>.70).

**Fig. 3:**
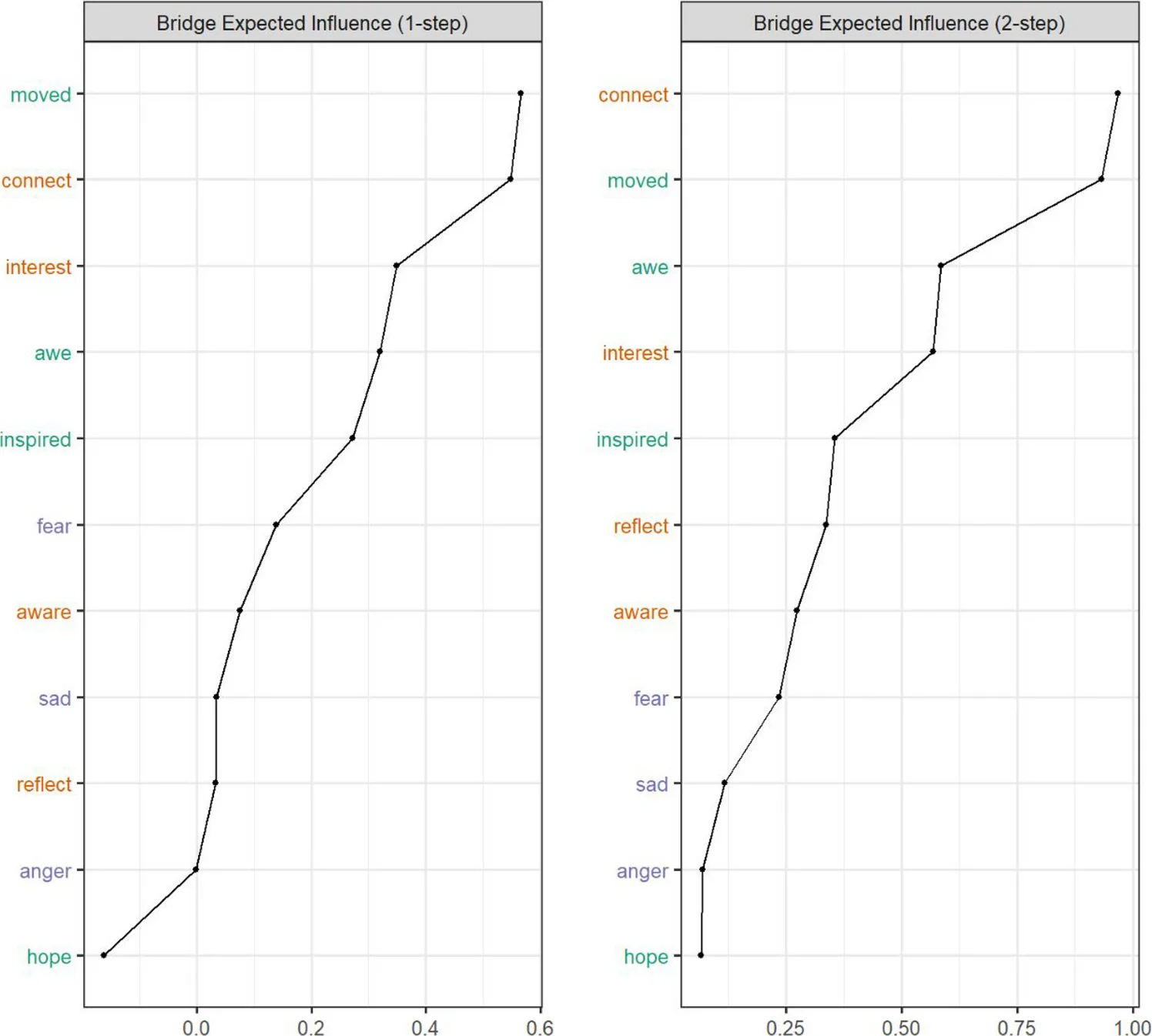
Standardized Estimates of Bridge Expected Influence (BEI) Indices (Steps 1 and 2) for Each Emotion Label in the Network. Note. Emotion labels are abbreviated: *moved* = moved/touched; *hope* = hopeful/optimistic; *awe* = awe/amazement; inspired = inspired/elevated; *interest* = interested/curious; *fear* = scared/afraid; *sad* = sad/unhappy; *anger* = angry/outraged; *connect* = emotional connection to the theme; *reflect* = reflection on the theme; *aware* = awareness of the theme

Finally, the bridge expected influence (BEI) estimated the extent to which each emotion maintains direct connections between dimensions (Step 1) but also indirect links through intermediary nodes (Step 2). As shown in Fig. 3, the highest bridge values were observed for emotions in the *self-transcendent* and *epistemic* dimensions, particularly for *moved* (BEI_step1 =.57; BEI_step2 =.93) and *connect* (BEI_step1 =.55; BEI_step2 =.97), indicating that these emotions not only exhibit the strongest direct links across dimensions, but also demonstrate indirect relations with other emotions throughout the network. Together, these results suggest that *moved* and *connect* function as central emotional hubs, linking positive self-transcendent and epistemic emotions and, to a lesser extent, also connecting them with negative responses.

## Discussion

To our knowledge, no existing dataset of street art images includes emotional ratings. Most available databases focus on other types of images, with little emphasis on art, and even less on street art. Although some studies have used street art images, these were selected specifically to serve their research aims (e.g., Gartus & Leder, 2014; Vessel et al., 2012, 2013, 2014).

Overall, our study shows that street artworks predominantly elicited pleasurable feelings and moderate arousal in participants. They also engaged viewers cognitively, with the most intense responses including reflection, awareness, and emotional connection to the artwork’s subject, as well as interest and being moved. Awe, inspiration, and hope were also experienced at moderate levels. In contrast, negative emotions such as fear, sadness, and anger were less experienced across most images in the dataset.

Most previous studies with image datasets with varied content tend not to consider multilevel analyses that account for variability introduced by participants and images, often treating all observations as independent. This approach may obscure important sources of variance. In contrast, our work uniquely provides adjusted emotional estimates at both the image and participant levels, derived from multilevel models that explicitly partition variability across stimuli and participants. Our analyses showed that participant-related factors significantly predicted emotional responses to artworks, particularly for positive emotions, with the highest proportion of variance observed for reports of interest and awe, while lower proportions were found for valence, arousal, and negative emotions. Additionally, variance attributable to participants exceeded that attributable to images for positive emotions and arousal. These results are consistent with recent computational findings showing that visually evoked affective responses to artworks are difficult to predict from low-level perceptual features (Conwell et al., 2025), highlighting the greater role of interpretative processes in aesthetic emotion elicited by street art (Stamkou et al., 2024; Vessel et al., 2013, 2014). Vessel et al. (2014), for example, showed that aesthetic experience in art is largely shaped by subjective, individual-level factors**.** While their work focused on inter-observer agreement in aesthetic preference judgments, our findings extend this account by showing that positive aesthetic emotions and arousal are also strongly individualized. Nevertheless, image-level random effects remained relevant for most emotions. In particular, for valence and negative emotions, images elicited more consistent responses than participant-related factors, suggesting that they remain more closely tied to shared, stimulus-based appraisals elicited by stimuli that convey social, economic, or environmental threats. These results are consistent with appraisal theories, which propose that negative stimuli activate shared evaluative processes related to relevance, harm, and norm violation, thereby constraining individual interpretation and reducing variability in emotional responses (Ellsworth & Scherer, 2003; Scherer, 2009). This interpretation also aligns with moral intuition models, which propose that social situations involving harm, unfairness, or norm violations are rapidly appraised as positive or negative, leading to strongly valenced emotional responses (Haidt & Joseph, 2004).

### Predictors of emotional responses

To further explore the participant-related factors, we considered nationality, age, gender, prior interest in street art (ISA), and sustainability consciousness (SC). Among these, ISA emerged as the most consistent predictor, explaining both valence and all positive emotions, indicating that a stronger appreciation for street art elicits greater pleasure and cognitive engagement, which may make us more receptive to its communicative aims. This pattern aligns with previous findings, indicating that higher ISA is associated with stronger emotional and cognitive evaluations of artworks (Gartus & Leder, 2014; Gartus et al., 2015). Additionally, the lack of association between ISA and negative emotions may be explained by the specific themes of the images that triggered these emotions, which often convey warnings about the negative environmental, social, and economic consequences of the depicted themes. Such content may exert a similar impact on viewers regardless of their interest in this art form, leading to more consistent negative emotional responses.

Sustainability consciousness (SC) also played an important role in predicting stronger *arousal,* most *positive emotions,* and *sadness*, suggesting that positive attitudes and behaviors toward social and environmental sustainability may shape how viewers emotionally engage with street artworks addressing these themes. The exception was feeling *awe*, consistent with the idea that it often arises from aesthetic qualities of the artwork itself (Keltner & Haidt, 2003), rather than from the sustainability-related content it may convey.

Differences between nationalities also emerged, with Brazilian participants reporting stronger feelings of being moved and greater awe. While previous research found that Brazilians typically report higher valence and arousal than Portuguese participants when viewing affective images (Soares et al., 2015), this pattern did not appear in our study. Cultural differences in emotional responsiveness may depend on the type of visual stimuli and may not generalize to street artworks. The affective impact of these artworks seems to be relatively consistent across gender and age. However, these findings refer to the responses across the full set of images rather than to specific themes or artworks. Gender differences in aesthetic and emotional processing remain inconclusive in the literature. For instance, Cela-Conde et al. (2009) found no gender differences in the frequency or subjective ratings of beauty appreciation, although such differences may depend on the thematic content of the images (Deng et al., 2016). Age may also differentiate responses to visual art in nuanced ways. Some studies have reported a “positivity bias” that emerges with age, reflecting an increased focus on positive content and a more lenient interpretation of negative displays (Branco et al., 2023). Future research could explore whether particular themes might evoke differentiated emotional responses across gender and age groups (e.g., gender equality, education). For example, artworks depicting women, children, older adults, or educational contexts, whether conveying optimistic, prosocial messages or more rebellious, provocative themes, may resonate differently depending on demographic backgrounds and life experiences.

We identified only a few predictors of valence, arousal, and negative emotions. ISA was associated with valence, whereas SC predicted arousal and sadness. However, the remaining predictors showed negligible associations, which may reflect the characteristics of the street art stimuli selected, the operationalization of emotions, the nature of the predictor variables, potential response biases, and the inherently multifaceted and subjective nature of emotional reactions to art. We should highlight that most participants did not report intense negative emotions, consistent with prior work showing a stronger positive evaluation of visual art (e.g., Achlioptas et al., 2021). Nevertheless, these low ratings may have contributed to a floor effect, reducing variance and limiting the detection of associations. Also relevant, while ISA was positively related to positive emotions, it did not predict negative emotions, suggesting that participants might have been less prone to express negative emotions toward street art, because our selection of artwork included works aligned with the themes of the United Nations Sustainability Development Goals (SDGs), which may have fostered more positive appraisals and mitigated negative emotions.

### Interrelations between affective responses

Our study also investigated how affective responses are interrelated to determine whether emotions align with the structure proposed by core affect models (Russell & Carroll, 1999)*,* or reflect a more multidimensional emotional landscape. Based on the affect circumplex model (Russell & Carroll, 1999)*,* affective experiences are organized along valence and arousal, with positive–negative affect and high–low arousal located at opposite ends of the underlying continua. However, our findings suggest that this framework only partially captures the affective responses elicited by the street art images.

First, the linear association between valence and arousal was very small and was classified as negligible by the AH test. Although the quadratic model suggested a shallow curvilinear pattern, broadly compatible with previous work showing that arousal can increase toward the extremes of valence in affective images, valence’s overall explanatory contribution to arousal remained limited. This result is broadly in line with Kuppens et al. (2013), who found a weak asymmetric V-shaped relation between valence and arousal in responses to nonfigurative modern art paintings, with sizable individual differences in the observed relations. Accordingly, this interpretation should be approached with caution, as the valence–arousal curvilinear relationship appears to be weak and may vary across contexts and cultures (Kuppens et al., 2013; Yik et al., 2023).

Second, the associations between valence and the other emotion labels revealed a differentiated pattern. Inspiration and hope showed a predominantly positive linear association with valence, reflecting their positive uplifting and optimistic tone. Sadness, fear, and anger showed predominantly negative linear associations with valence, consistent with their negative affective polarity. In contrast, moved, awe, reflection, awareness, connection, and interest suggested modest curvilinear patterns with valence, suggesting that these responses may be elicited more strongly by images at both positive and negative ends of the valence continuum, although the strength of this result varied across emotional responses. Overall, these findings indicate that emotional responses to street art cannot be reduced to positive–negative polarity. Instead, some responses may involve both pleasant and unpleasant feelings, which are not fully captured by a bipolar valence dimension that requires a single evaluative position (Stamkou et al., 2024).

Third, the linear associations between arousal and emotion labels were small and classified as negligible by the AH tests. Overall, the image-level estimates were concentrated within a relatively narrow range of moderate arousal. The restricted range may help explain the limited associations between arousal and the affective responses. Also, the moderate arousal may reflect the type of engagement elicited by the street art images, since the strongest responses were observed for reflection and awareness, suggesting engagement primarily at an interpretative and thematic level, involving the appraisal of meaning, relevance, novelty, and complexity, but without necessarily producing either calm or high subjective activation. This interpretation is consistent with evidence suggesting that socially emotional stimuli require more elaborative and context-dependent interpretation, which can be less directly associated with strong arousal (Sakaki et al., 2012). Moreover, when arousal was modeled as an outcome, the variance was more strongly clustered at the participant level than at the image level, indicating that felt arousal may depend more on viewer-level characteristics than on differences between images. Consistent with this interpretation, felt arousal was slightly higher among participants with greater sustainability consciousness, suggesting that it may depend in part on viewers’ sensitivity to the themes depicted.

The network analysis showed a richer pattern of interrelations among the emotion labels, including the positive links between positive and negative emotions when engaging with street art, a finding consistent with the paradox of deriving positive affect from negative content in artistic contexts (Bantinaki, 2012; Goldstein, 2009; Wagner et al., 2016).

Overall, we identified a three-dimensional structure of specific emotion labels with stability and structural consistency: *Self-transcendent*, encompassing emotions such as *moved*, *awe*, *inspired*, and *hope*, reflected the inspiration, moral elevation, and kama muta often associated with aesthetic appreciation of art in general, and with recognizing communal shared relations, humanity, and moral beauty (Abitbol & VanDyke, 2023; Clayton et al., 2021; Oliver et al., 2018; Pizarro et al., 2021; Zickfeld et al., 2019). The second, termed *epistemic*, includes interest, reflection, awareness, and connection with the themes depicted, capturing emotionally infused forms of cognitive engagement and meaning-making in response to the artwork and its themes, often arising when individuals encounter novelty, incongruity, complexity, gaps in understanding, themes demanding interpretation, and cognitive resolution (Brun et al., 2008; Kashdan & Silvia, 2009; Morton, 2010; Nerantzaki et al., 2021; Schindler et al., 2017; Vogl et al., 2021). The third dimension, *negative emotions*, comprised anger, fear, and sadness, which tend to arise when people are confronted with images depicting threats, moral concerns, suffering, or injustice. For example, children in contexts of vulnerability or violence, such as wounded children behind barbed wire (image 776), or a child facing a lineup of rifles labeled with the names of schools affected by shootings (image 701), were among those eliciting the strongest responses on negative emotions. These emotions appear to reflect an affective system oriented toward social concern and moral agency in response to the artworks’ critical messages (Batson et al., 2007; Iyer et al., 2007; Nesse & Ellsworth, 2009; Silvia & Brown, 2007; Tannenbaum et al., 2015).

The analysis of bridge centrality further showed that moved and connect occupied central positions in the network, linking the *self-transcendent* and *epistemic* dimensions and connecting to emotions within the *negative* cluster. Specifically, being moved showed the highest bridge influence, maintaining positive connections with both positive and negative emotions. Several artworks that elicited high levels of moved also scored strongly on sadness, reflection, and awareness. For example, image 775, which depicts children sitting on the ground with empty bowls behind barbed wire, received particularly high ratings for awareness, followed by reflection, sadness, and moved. These artworks illustrate how moved can also be linked to epistemic emotions and sadness, especially when the stimulus foregrounds vulnerability or morally human concerns. This pattern is also consistent with prior studies showing that being moved can occur in conjunction with sadness and often evokes meaning-making (Schindler et al., 2022). Similarly, emotional connection with the themes showed bridging links not only to self-transcendent emotions such as inspiration and hope, but also with fear and anger (for example, image 697 addressing child abuse elicited high anger but also strong emotional connection, which may reflect moral outrage intertwined with empathy). In contrast, artworks depicting scenes of social equality evoked emotional connection, inspiration, and hope, reflecting positive, socially constructive forms of connection (for example, images 178 and 249, which portray women from diverse cultural backgrounds engaged in different professions or in educational contexts). Together, these patterns highlight the interconnected nature of emotions evoked by street art and the coexistence of emotions of opposite valence, demonstrating that it can simultaneously provoke distinct, even contrasting, emotions in response to a single artwork (Stamkou et al., 2024).

Our dataset revealed considerable variability in emotional responses across participants and images. Overall, positive emotions elicited by street artworks were generally more intense and displayed greater inter-individual variability, reflecting the diverse personal and contextual factors shaping viewers’ experiences. As already mentioned, such variability is consistent with research showing that emotional responses to visual art and complex themes vary widely across individual interpretations (Conwell et al., 2025; Stamkou et al., 2024; Teh et al., 2018; Vessel et al., 2014). This dispersion likely stems from the multifaceted content of many artworks, which combine aesthetic, social, economic, and environmental issues. For example, some images juxtapose distressing and hopeful elements (e.g., image 188 depicting a baby held by a protective hand alongside text referencing the number of children born with AIDS). Viewers may also attend to different features of the artworks, resulting in divergent emotions that reflect the layered meanings of the images rather than a single emotional theme. Consequently, mid-range valence ratings may not signify neutrality but rather ambivalence, a pattern previously observed in responses to morally and aesthetically complex art (Teh et al., 2018). In contrast, negative emotions were less intense yet more consistent across participants, likely reflecting a common response to the alarming social, economic, and environmental themes depicted in the dataset. Thus, when artworks depict salient threats, affective responses may be guided by evaluative mechanisms that are more similar across individuals, resulting in greater inter-individual convergence in valence and negative emotions (Ellsworth & Scherer, 2003; Haidt & Joseph, 2004; Scherer, 2009).

Together, these findings suggest that negative emotions reflect shared awareness of critical issues, whereas positive emotions capture greater diversity and subjectivity in responses to the street art dataset, underscoring the need for future studies to account for individual differences and to examine additional predictors of both positive and negative emotional responses. Our findings also have implications for how emotional data from this dataset should be interpreted and used. The street artworks are diverse, and the emotional dataset provides valuable information on how people respond to street art in general; however, given the overall variability, researchers should further examine how specific visual or thematic content relates to consistent emotional patterns. Selecting artwork based on content and focusing on emotions that show greater consistency across image subsets may help identify homogeneous groups that yield more generalizable emotion patterns in future research.

### Limitations and suggestions for future studies

Although our study offers a new dataset of street art and novel insights into emotional responses to these images, several limitations should be acknowledged.

We used photographs of street artworks, which are not reproductions of the original artworks but mediated representations influenced by the photographer’s choices (e.g., framing, angle, lighting). The images also captured street artworks at a specific moment in time and space, representing a transient form of artistic expression that may have changed or disappeared. As street art often responds to contemporary events, some themes depicted may no longer carry the same impact as when they were first created (e.g., tribute to firefighters involved in the 9/11 attacks [image 313] or to healthcare workers painted during the COVID-19 pandemic [images 393; 309; 310]). Consequently, the meanings and emotional intensity of specific artworks may reflect shifts in collective memory and sociocultural relevance.

The way the artworks were viewed online may also have introduced variability, as all images were presented online, and their visual dimensions depended on each participant’s screen size, resolution, and display settings. Many images also contain the artwork itself rather than its immediate surroundings, despite recognizing that contextual elements are integral to interpreting street art (e.g., location, scale, environment, social or historical setting) (Gonçalves & Milani, 2022), and that artworks are embedded in the social and cultural contexts of the communities where they were created, illustrating local concerns, histories, and identities. This decision was aimed at minimizing the influence of extraneous variables on participants’ responses. Nevertheless, we also have images with elements of their environments, such as architecture, urban decay, or community life, which add layers of meaning, and artworks conveying sociopolitical messages relevant to their place of origin (e.g., images 494–499 of the Syrian artist Abu Al-Shami, which depict war, loss, resilience, and the search for hope amid conflict). These variations in artistic traditions, symbolism, and historical context may limit some viewers’ ability to fully grasp the meanings embedded in the artworks, potentially leading to misinterpretations or a reduced emotional impact. Nevertheless, the inclusion of this diversity aimed to capture a wide range of artistic styles and perspectives, enriching the dataset and facilitating future cross-cultural research.

The StreetArt4Sustainability dataset is also unbalanced in its geographical distribution of artworks, with some regions overrepresented. However, we expect the dataset to remain adaptable, enabling the inclusion of artworks from diverse cultural contexts to further explore the nuances of street art across nations. Such inclusivity may lead to a more holistic understanding of the global street art movement, the sociopolitical themes and narratives prevalent in unrepresented nations, and its reception across different cultures and societies. Relatedly, our data were collected in only two countries and via convenience sampling, limiting the generalizability of our findings to broader populations. Future studies should consider diversifying the participant pool.

Another limitation concerns the unequal number of affective ratings per image, as some images received fewer responses because the image set was adjusted during data collection and full balance across images was not achieved. Although the multilevel models accommodated this structure, images with fewer observations may have less precise image-level estimates. Future studies could extend the evaluation of this dataset with a more balanced number of ratings per image.

Given that not all pieces may have been interpreted as the artist initially intended, future studies could also provide contextual information about each artwork, such as the artist’s stated intent, the location, and the sociocultural context in which the piece was created, to help participants better situate their interpretations. Research has shown that additional contextual cues (e.g., titles, descriptions) can enhance viewers’ understanding of artworks, particularly for abstract or conceptually demanding pieces (Gerger & Leder, 2015; Leder et al., 2006). Viewing artworks online also differs substantially from experiencing them in situ. The loss of depth, scale, and color accuracy, as well as spatial immersion and the possible absence of environmental cues, may alter viewers’ perceptual and emotional engagement with the artworks (e.g., Gartus & Leder, 2014; Konečni, 2015).

Although our study included a broader range of emotion labels than most existing affective image datasets, the set of responses assessed remains restricted. Moreover, the emotion labels were mainly positive, which may have further introduced a positive bias and reduced the sensitivity to identify negative responses. Future research could aim for a more balanced representation of positive and negative emotions and also expand the set of labels to include more nuanced or ambivalent states relevant to street art, such as disgust (in response to moral or aesthetic violation), confusion (when the artwork’s meaning is obscure), indifference (reflecting emotional disengagement), or disappointment (when expectations are unmet) (Silvia, 2010; Silvia & Brown, 2007). Future studies could also examine affective responses associated with humorous, ironic, or satirical street art, such as mirth or amusement, since humor and satire may function as aesthetic and communicative strategies through which artworks challenge dominant discourses, which in turn may reduce defensiveness and promote reflection on social issues (Sørensen, 2017; Tunali, 2020).

Reliance on self-report measures is also a limitation, as they capture only the subjective component of emotion, whereas emotions are multifaceted, involving behavioral, cognitive, expressive, motivational, and neuropsychophysiological processes. Future studies could incorporate additional indicators of emotions, as employed in other affective stimulus datasets (e.g., Clayton et al., 2021), to advance understanding of participants’ emotions in response to street art. Self-report may also be influenced by social desirability or differences in emotional awareness, and emotion labels may vary in meaning across individuals and cultures (Barrett, 2006).

Additionally, since our focus was on the feelings elicited by the images, our study did not assess aesthetic attributes and image properties, such as visual complexity, or appreciative judgments, such as aesthetic value, beauty, or liking (e.g., Amirshahi et al., 2015; Fekete et al., 2023; Mallon et al., 2014), which can be relevant to assess in future studies.

Finally, future studies could integrate additional individual dispositions that may predict viewers’ responses to street art. For example, dispositional self-transcendence may increase susceptibility to awe and moral elevation (Yaden et al., 2017), whereas dispositional fear, sadness, or anger may increase attention to threatening information, which may in turn amplify emotional reactions to these specific emotion-eliciting artworks (Parsafar & Davis, 2018).

## Conclusion

The StreetArt4Sustainability dataset is the first collection of street art images rated for both dimensional and specific affective responses, enabling the study of how visual art evokes distinct, mixed, and self-transcendent emotions that are often overlooked in traditional affective image databases. In line with other validated stimulus sets (see the compiled work of Diconne et al., 2022), the dataset can be used to address a wide range of research questions, from studies about perception, attention, memory, and psychoneurophysiology (e.g., Bradley et al., 2001a, 2001b; Clayton et al., 2021) to computational modeling of how visual and semantic features predict affective responses (e.g., Conwell et al., 2025; Zhang et al., 2025). By including artworks from diverse cultural contexts and covering a wide range of themes, many of which were selected to be aligned with the United Nations SDGs, the dataset also opens new avenues for examining how visual art engages people with social and environmental issues, advancing research at the intersection of aesthetic emotions and sustainability.

## Data Availability

The StreetArt4Sustainability dataset and corresponding information are available at https://doi.org/10.5281/zenodo.17702005 for research purposes. The data collected from participants are available at https://doi.org/10.5281/zenodo.17966026. The website https://www.patriciaarriaga.site/streetart4sustainability allows interactive exploration of the images and raw aggregated affective responses.

## Code availability

The analysis code in R is available at https://doi.org/10.5281/zenodo.17966026. The GitHub repository (https://github.com/doomlab/StreetArt4Sustainability) also provides the source code and documentation for the dataset’s interactive visualization.

## Declarations

### Open practices statement

This study follows open science practices: the stimuli, aggregated and participant-level data, analysis code, and interactive materials are available in public repositories, and the research was preregistered; details are provided in the article and in the sections below.

### Preregistration

The research project was preregistered in AsPredicted (https://aspredicted.org/hhzc-7h8v.pdf), which included details on the dataset creation and two planned studies. The present manuscript focuses exclusively on Study 2. Space limitations prevented the inclusion of Study 1, which will be submitted separately because it has an independent focus (classifying images according to the SDGs). Some deviations occurred: (1) The preregistration specified a dataset of 400 street art images, whereas the final dataset was expanded to 556 images (this extension was implemented before the analyses were conducted). (2) The preregistration anticipated a target sample of 800 participants, whereas the recruitment was extended to 1,610 participants, with the final analytic sample comprising 1,239 participants after applying exclusion criteria. As a result, each image was evaluated by between 13 and 40 participants (*M* = 22). (3) The preregistration specified that results with *p* <.05 would be considered statistically significant, but the large sample size led to several statistically significant effects of negligible magnitude. To address this issue, we applied the Anderson–Hauck (AH) test to assess whether effects were non-negligible, using the smallest effect size of interest (SESOI) informed by prior normative affective datasets. (4) The preregistration specified exploratory correlational analyses among the emotions elicited by the images. During revision, in response to a reviewer’s suggestion, we extended these exploratory analyses by adding quadratic models examining the relation between valence and the emotion labels, together with a theoretical rationale for these additional analyses.
